# Sox5 controls the establishment of quiescence in neural stem cells during postnatal development

**DOI:** 10.1371/journal.pbio.3002654

**Published:** 2025-07-28

**Authors:** Cristina Medina-Menéndez, Paula Tirado-Melendro, Lingling Li, Pilar Rodríguez-Martín, Elena Melgarejo-de la Peña, Mario Díaz-García, María Valdés-Bescós, Rafael López-Sansegundo, Aixa V. Morales

**Affiliations:** Instituto Cajal, CSIC, Madrid, Spain; University of Cambridge, UNITED KINGDOM OF GREAT BRITAIN AND NORTHERN IRELAND

## Abstract

Adult stem cell niches relays in the acquisition of a reversible state of quiescence to ensure long-lasting DNA integrity and cell expansion. Neural stem cells (NSCs) in the dentate gyrus (DG) enter quiescence before the adult hippocampal neurogenic niche is fully established. However, the mechanisms controlling NSC first quiescence entry and quiescence deepness are largely unknown. Using conditional mutant mouse during embryonic or postnatal stages, we have determined that transcription factor Sox5 is required to restrict first entry in quiescence. Moreover, we have found a critical window during the second postnatal week when NSCs build up a shallow quiescent state. Loss of Sox5 leads to an excess of NSCs in shallow quiescence, which are prone to activate, leading to a neurogenic burst in the adult DG and precocious depletion of the NSC pool. Mechanistically, Sox5 prevents an excess of BMP canonical signaling, a pathway that is required to maintain the correct levels of NSC quiescence during the second postnatal week. In conclusion, our results demonstrate that Sox5 is required to control the correct balance between shallow and deep quiescence during the first postnatal weeks of DG development, a balance which is essential for establishing long-lasting adult neurogenesis.

## Abstract 2

In the hippocampus, neural stem cells (NSCs) enter quiescence during postnatal development, forming a pool of stem cells that support neurogenesis in adulthood. This study shows that NSCs acquire different levels of quiescence and that the transcription factor Sox5 controls the balance between shallow and deep quiescence during hippocampal development.

## Introduction

During embryonic and postnatal development of the dentate gyrus (DG), neural stem cells (NSCs) proliferate, migrate, and generate mature granule neurons (GN) and astrocytes. In sharp contrast to other brain regions, in the subgranular zone (SGZ) of the adult DG, a subpopulation of NSCs remains in a reversible quiescent state (qNSCs) [1–4]. Through highly regulated molecular mechanisms, those adult NSCs are activated (aNSCs) and enter in cell cycle to produce intermediate progenitor cells (IPCs) that generate new GNs in the DG throughout adult life [5,6].

NSCs in the DG enter in a quiescence state predominantly during the first postnatal week, and a high percentage of them will remain as a source of adult new neurons [1–3]. Recently, it has been shown that autophagy drives the conversion of developmental NSCs to the adult quiescent state [7]. However, little is known about the transcriptional program controlling this first quiescence entry and how the reversibility of the initial quiescent state is achieved.

Furthermore, quiescence is not an unique static state defined by cell cycle absence. Single-cell RNA-Seq analysis of adult NSCs support the idea of a continuum of cell states or NSC populations from a deep/dormant quiescent state to an activated state [8–10]. This includes a shallow/resting/primed quiescent state defined, in comparison with the dormant state, by higher expression of cell cycle genes, upregulation of ribosomal genes and a shift in energy metabolism genes [8–11]. Moreover, the primed state can be captured in culture where combinations of cell cycle transcripts define primed NSCs at a half-way position between qNSCs and aNSCs [12,13]. However, it is unclear how NSCs acquire this shallow quiescence state as they enter quiescence and convert into adult NSCs during the first postnatal weeks.

Despite the complexity of signaling pathways acting within the NSC adult niche, candidates for promoting adult NSC quiescence have emerged. These include BMP4 [14,15] and its downstream effectors Id1 and Id4 [16–18]; TGFβ [19]; Delta/Notch pathway [20–22] and the MFGE8/integrin/ILK pathway [23], amongst others. Nevertheless, we know very little about signals and transcription factors that could modulate quiescence during DG development.

Now, using mouse conditional mutant for Sox5 during embryonic or early postnatal development, we describe a critical window around P14 when NSCs build up a shallow quiescent state. During that period, Sox5–defective mice exhibit an increase in shallow qNSCs that leads to severe alterations in the adult DG neurogenic niche, including transient aberrant NSC activation and excessive neurogenesis. As a consequence, older *Sox5* mutant mice show a premature reduction in the adult NSC pool and in neurogenesis. Mechanistically, Sox5 prevents an excess of pSmad1/5/9/Id4 and cell cycle genes expression during the second postnatal week. Moreover, we show that correct levels of BMP canonical signaling pathway have to be tightly regulated to prevent an excess of NSCs in a shallow quiescence state that would compromise the long-lasting nature of the adult neurogenic niche.

## Results

### NSC population and proliferation decrease drastically during the second postnatal week of DG development

**Fig 1:**
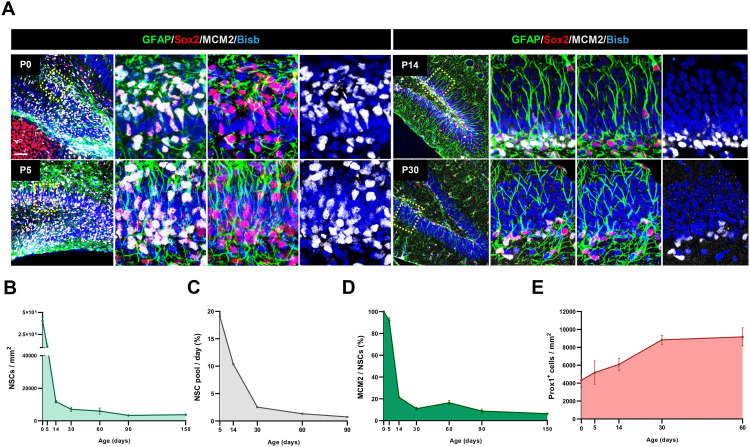
NSC population and proliferation decrease drastically during the second postnatal week of DG development. **(A)** Confocal images showing proliferative NSCs (rGFAP^+^Sox2^+^MCM2^+^) in the DG at the indicated mice stages. **(B)** Quantitation of NSCs/mm^2^ in the DG (rGFAP^+^Sox2^+^ cells) at different stages. **(C)** NSC depletion rate per day in DG. **(D)** Proliferating NSC quantitation in the NSC population (%). **(E)** Quantitation of Prox1^+^cells per mm^2^. At least three animals and three sections/animal were analyzed for each immunostaining. In all graphs, data are mean value ± SEM. ***p < 0.001 by unpaired Student *t* test. Scale bars represent 30 µm (A, right panels), 100 µm (A left panels). Data are available in S1 Data as a part of Supporting information.

During the first postnatal week the majority of NSCs in the developing DG enter quiescence for their first time [1–3] and most adult GNs are generated [24,25]. In order to systematically quantitate possible changes in the NSC population during postnatal development, we identified NSCs by GFAP and Sox2 expression and determined NSC proliferating fraction using MCM2 (a cell cycle marker) from P0 to P150 (Fig 1A–1D). We observed a dramatic NSC loss during the first postnatal week (P0 > P5) from 4.16 × 10^6^ to 1.1 × 10^6^ cells/mm^2^ (Fig 1A and 1B) accounting for a depletion rate of 19.9% of NSCs per day (Fig 1C). However, we observed that NSC proliferation rate (% of MCM2^+^ NSCs) remained stable during that period (Fig 1D). The reduction in the NSC pool during P0 > P5 interval could be due to the peak in GN differentiation previously described [24,25] and supported by the increase in Prox1^+^ GNs from 4329 ± 819 to 5187 ± 1306 cells/mm^2^ (Fig 1E).

By the second postnatal week, NSC loss continued (from 1.1 × 10^6^ to 11846.5 cells/mm^2^ at P5 > P14; Fig 1A and 1B), albeit at a slightly lower depletion rate (10.4% daily loss; Fig 1C). Nevertheless, during this second week, we observed a dramatic loss of the NSC proliferating fraction (only 21.5 ± 0.9% of the NSC pool are proliferating by P14; Fig 1D), whereas Prox1^+^ cells increased to 6093 ± 692 cells/mm^2^. These data suggest that by the end of the second postnatal week the depletion of the NSC pool could be due both to NSC differentiation and to NSC accumulation in a quiescent state that prevents new rounds of NSC self-renewal.

At early adulthood, the pool of NSCs declined sharply by P30 at a rate of 2.6% per day and at a moderate rate by P60 and P90 (Fig 1A–1C). In parallel, the proliferating fraction halved (10.9 ± 1.4% of MCM2^+^ NSCs; Fig 1A,1D) with respect to that in P14 and then remained similar by P60 and P90 (16.4 ± 2.1% and 8.8 ± 1.9%; Fig 1D). This coincides with the increase in Prox1^+^ GNs density by P30 and P60 (up to 9168 ± 1011 cells/mm^2^; Fig 1E).

Thus, there are two temporal windows of high NSC variation: (i) P0 > P5, when the NSC pool is severely reduced mostly due to massive neuronal differentiation and (ii) P5 > P14, when the NSC pool decreases possibly due to both cell differentiation and massive NSC accumulation in quiescence.

### Sox5 is required to restrict NSC entry in quiescence during the first postnatal week

**Fig 2:**
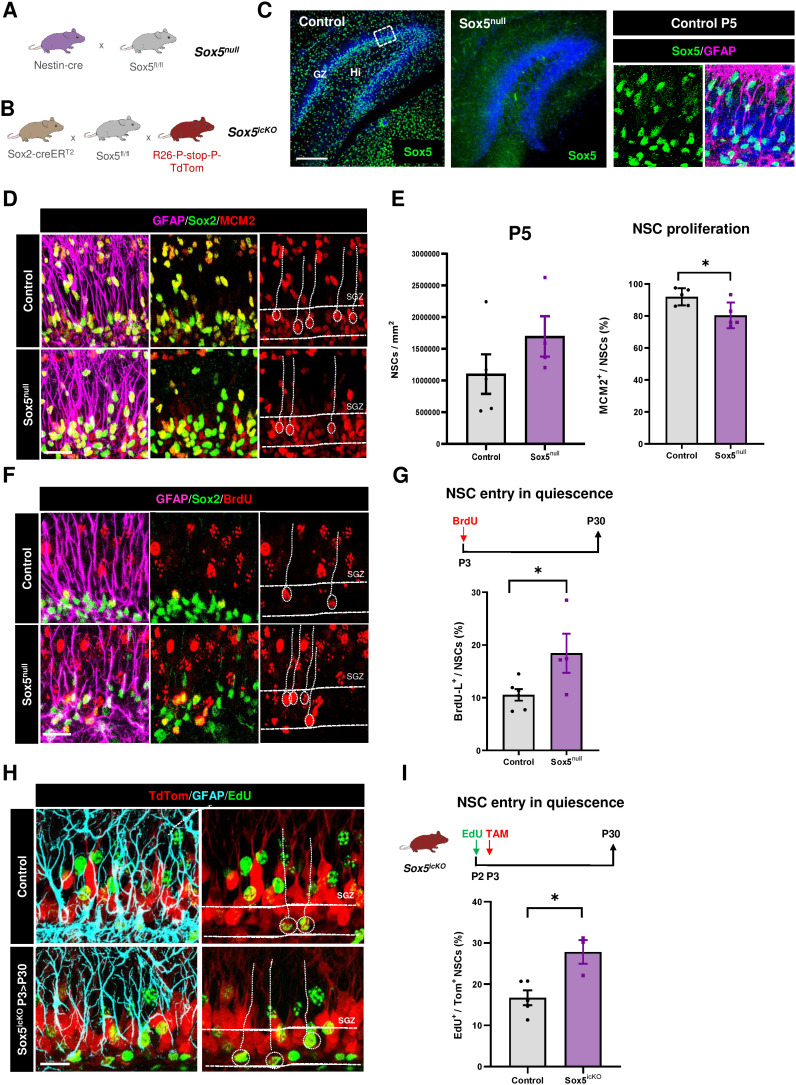
Sox5 is required to restrict NSC entry in quiescence during the first postnatal week. **(A, B)** Generation of Sox5^null^ (A) and Sox5^icKO^ (B) mice. **(C)** Confocal images showing Sox5 expression in dorsal DG of Control and Sox5^null^ mice by P5 and Sox5^+^ cells expressing GFAP in Control P5 DG. **(D)** Confocal images showing GFAP, Sox2, and MCM2 immunostaining in the SGZ (space between white lines) of P5 Control and Sox5^null^ mice. **(E)** Quantitation of NSCs/mm^2^ (rGFAP^+^Sox2^+^ cells) and proliferative MCM2^+^ NSCs (rGFAP^+^Sox2^+^MCM2^+^ % over total NSCs) in the SGZ of Control and Sox5^null^ P5 mice. **(F)** Confocal images showing GFAP, Sox2, and BrdU immunostaining in P30 Control and Sox5^null^ mice after P3 BrdU pulse. **(G)** Scheme of BrdU-long labeling retention experiment. Quantitation of the % of BrdU-L^+^ cells in NSCs (rGFAP^+^Sox2^+^) in P30 Control and Sox5^null^ mice. **(H)** Confocal images showing TdTom, GFAP, and EdU immunostaining in the DG of P30 Control and Sox5^icKO^ mice. **(I)** Scheme of EdU-long labeling retention experiment in TAM injected conditional Sox5^icKO^ mice (P3 > P30). Quantitation of the % of EdU^+^ cells in TdTom^+^ NSCs of P30 Control and Sox5^icKO^ mice. Data represent mean values ± SEM. *p < 0.05, **p < 0.01, and ***p < 0.001 by unpaired Student *t* test. Horizontal dotted lines delineate the SGZ. Scale bars represent 100 µm (C) and 50 µm (D, F, H). Data are available in S1 Data as a part of Supporting information. Drawings were created through SciDraw.

In order to understand early dynamics in NSC population, we analysed the expression of transcription factor Sox5 that is required for the transition from quiescence to activation in the adult DG [26]. We observed that Sox5-expressing cells were widely distributed in the developing hilus and granular zone (GZ) of the DG from embryonic day 16.5 to P5 (Fig 2C and S1 Fig). At P5, NSCs are accumulated in the future SGZ and the majority of Sox5^+^ cells in the DG co-expressed NSC markers Sox2, GFAP, and Hopx and were proliferating (Figs 2C and S1C,S1D). Moreover, we estimated that 9.6 ± 0.8% of Sox2^+^ cells were Tbr2^+^ IPCs and almost none of them were Prox1^+^ GNs (S1C and S1E Fig). Considering the high level of Sox5 and Sox2 co-expression, we could assume that Sox5 was expressed in around 10% of IPCs and almost absent from GNs.

By P14, the majority of Sox5^+^ cells were radial (r) GFAP^+^/Sox2^+^ cells but more than half of them stopped proliferating (only 36.1 ± 6.1% were MCM2^+^) and only 57.2 ± 3.5% retained Hopx (S1C and S1D Fig). Hopx expression is progressively restricted to quiescent NSCs during postnatal DG development [27]. Thus, Sox5^+^Hopx^+^ cells could represent the increasing pool of quiescent NSCs by P14. Further analysis showed that in the Sox2^+^ cell population, 8.2 ± 0.9% of cells were Tbr2^+^ IPCs and 3.5 ± 1.7% were Prox1^+^ GNs (S1C and S1E Fig). In conclusion, Sox5 expressing cells in the postnatal developing DG were predominantly NSCs and Sox5 expression decreases as NSCs progress along the neurogenic cascade.

To determine if Sox5 was required for NSC entry into quiescence for their first time, we resourced to a conditional Sox5^fl/fl^ line [28] crossed to a Nestin-cre line to remove Sox5 expression from neural progenitors starting around embryonic stage 13.5 (Fig 2A and 2C; Sox5^null^). During the first postnatal week, we observed that loss of Sox5 reduced NSC proliferation with respect to Control mice (80.4 ± 3.6% versus 92.0 ± 2.4% of MCM2^+^ cells in rGFAP^+^/Sox2^+^ NSCs; *P* = 0.029; Fig 2D and 2E). However, the NSC pool was similar in Sox5^null^ and Control mice (Fig 2E).

To explore if the reduction in aNSCs in Sox5^null^ mice was due to an abnormal entry in quiescence, we performed a single BrdU injection at P3 and chased NSCs by P30 (Fig 2G). Dividing NSCs can incorporate BrdU and retain it for as long as they remain quiescent **[**BrdU-long (BrdU-L) retaining NSCs]. By P30, the percentage of BrdU-L NSCs with respect to total NSCs was larger in Sox5^null^ than in Control mice (18.43 ± 3.7% versus 10.53 ± 1.1%, respectively; *P* = 0.041; Fig 2F and 2G). Thus, embryonic Sox5 loss provoked postnatal increase in the number of NSCs entering quiescence and a reduction in aNSCs.

As neural progenitors of the embryonic DG express Sox5 (S1A Fig), Sox5^null^ NSC defects at P5 could be due to embryonic alterations. To clarify this aspect, we resourced to a conditional inducible strategy using a Sox2-creER^T2^ mouse line crossed with Sox5^fl/fl^ and reporter Rosa-TdTomato lines previously characterized (Sox5^icKO^; Fig 2B) [26]. Moreover, this type of experiment allowed us to follow the analysis in a cell-autonomous manner and prevented limitations in EdU incorporation due to defects in proliferation. Thus, we observed that Sox5 loss induced by TAM injection at P3, preceded by a single EdU injection, caused a dramatic increase in recombined TdTom^+^ EdU-Long retaining NSCs by P30 with respect to Control mice (27.83 ± 2.87% versus 16.70 ± 1.81%, respectively, *P* = 0.0132; Fig 2H and 2I). As these results were similar to those observed in Sox5^null^ mice (Fig 2F and 2G), we concluded that Sox5 is required during the first postnatal week to prevent an excess of NSCs entering quiescence for their first time and thus to maintain NSCs in a proliferative state which ensures adequate NSC number.

### Embryonic loss of Sox5 reduces the pool of dormant quiescent NSCs by P14

**Fig 3:**
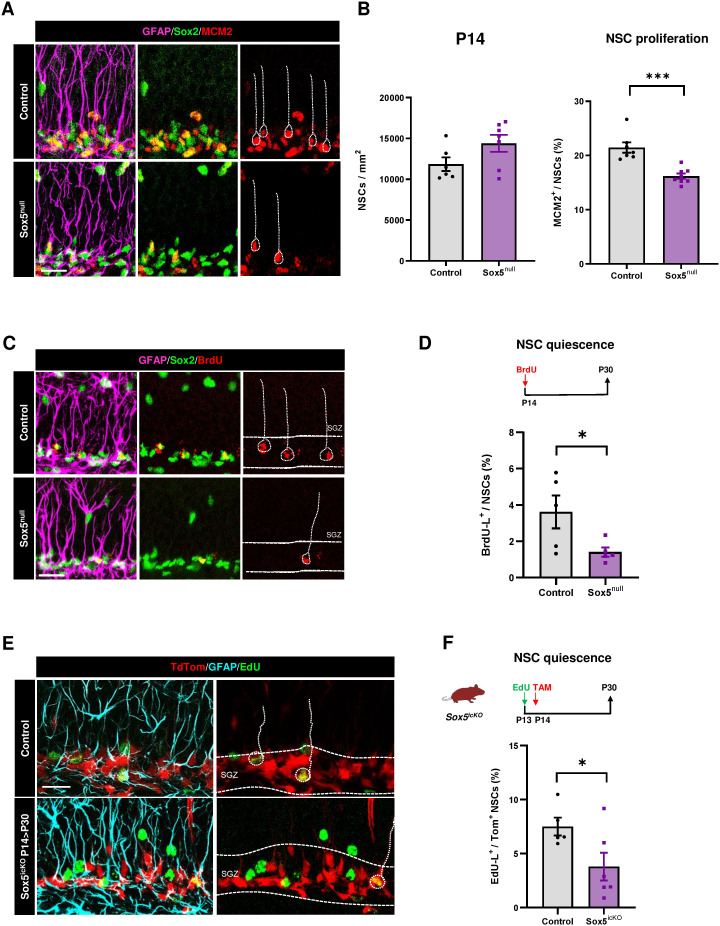
Embryonic loss of Sox5 reduces the pool of quiescent NSCs by P14. **(A)** Confocal images showing GFAP, Sox2, and MCM2 immunostaining in the SGZ (space between white lines) of P14 Control and Sox5^null^ mice. **(B)** Quantitation of NSCs/mm^2^ (rGFAP^+^Sox2^+^ cells) and proliferative MCM2^+^ NSCs (% over total NSCs) in Control and Sox5^null^ P14 mice. **(C)** Confocal images showing GFAP, Sox2, and BrdU immunostaining in P30 Control and Sox5^null^ mice after P14 BrdU pulse. **(D)** Scheme of BrdU-long labeling retention experiment. Quantitation of the % of BrdU-L^+^ cells in NSCs (rGFAP^+^Sox2^+^) of P30 Control and Sox5^null^ mice. **(E)** Confocal images showing TdTom, GFAP, and EdU immunostaining in the SGZ of P30 Control and Sox5^icKO^ mice. **(F)** Scheme of EdU-long labeling retention experiment in TAM injected conditional Sox5^icKO^ mice (P14 > P30). Quantitation of the % of EdU-L^+^ cells in TdTom^+^ NSCs of P30 Control and Sox5^icKO^ mice. In all graphs, data represent mean value ± SEM. *p < 0.05, **p < 0.01, and ***p < 0.001 by unpaired Student *t* test. Scale bars represent 50 µm (A, C) and 20 µm (E). Data are available in S1 Data as a part of Supporting information. Drawing was created through SciDraw.

To determine if Sox5 was required during the second postnatal week to maintain NSC proliferation, we analyzed Sox5^null^ mice by P14 and observed a similar reduction to that in P5 in aNSCs % with respect to Control mice (16.2 ± 0.5% versus 21.5 ± 1.0% of MCM2^+^ cells in the population of rGFAP^+^ Sox2^+^; *P* = 0.0002; Fig 3A and 3B). Moreover, the NSC pool did not change (Fig 3B). These results indicate that Sox5 is required for NSC proliferation during the first two weeks of postnatal DG development.

Unexpectedly, in Sox5^null^ mice injected with BrdU at P14 and chased by P30 we observed a reduction in BrdU-L^+^ NSCs (with respect to total NSCs) in comparison with Control mice (1.41 ± 0.25% versus 3.62 ± 0.9% of BrdU^+^ in rGFAP^+^ Sox2^+^ NSCs; *P *= 0.0466; Fig 3C and 3D). As NSCs in Sox5^null^ mice had a reduced level in proliferation that could have compromised BrU incorporation, we resourced to Sox5^icko^ mice to label aNSCs with Edu before Sox5 loss at P13 and then promote Sox5 loss by P14 > P30 by TAM injection. Similarly, to what we observed in P14 Sox5^null^, P14 > P30 Sox5^icKO^ mice exhibited a robust decrease in the % of EdU-L^+^ TdTom^+^ NSCs over recombined TdTom^+^ NSCs with respect to Control mice (3.8 ± 1.3% versus 7.5 ± 0.8% respectively, *P *= 0.0469; Fig 3E and 3F), indicating a decrease in NSCs quiescence maintenance in the P14-P30 period due to postnatal Sox5 loss.

In summary, these data indicate that around P14, Sox5 is required for NSC proliferation but also to maintain NSCs in a prolonged state of quiescence for at least 15 days. Moreover, these would suggest that there could be a subpopulation of NSCs, probably in an intermediate state of shallow quiescence, which is affected by Sox5 loss.

### Sox5 loss causes a general over activation of NSCs in young adult mice

**Fig 4:**
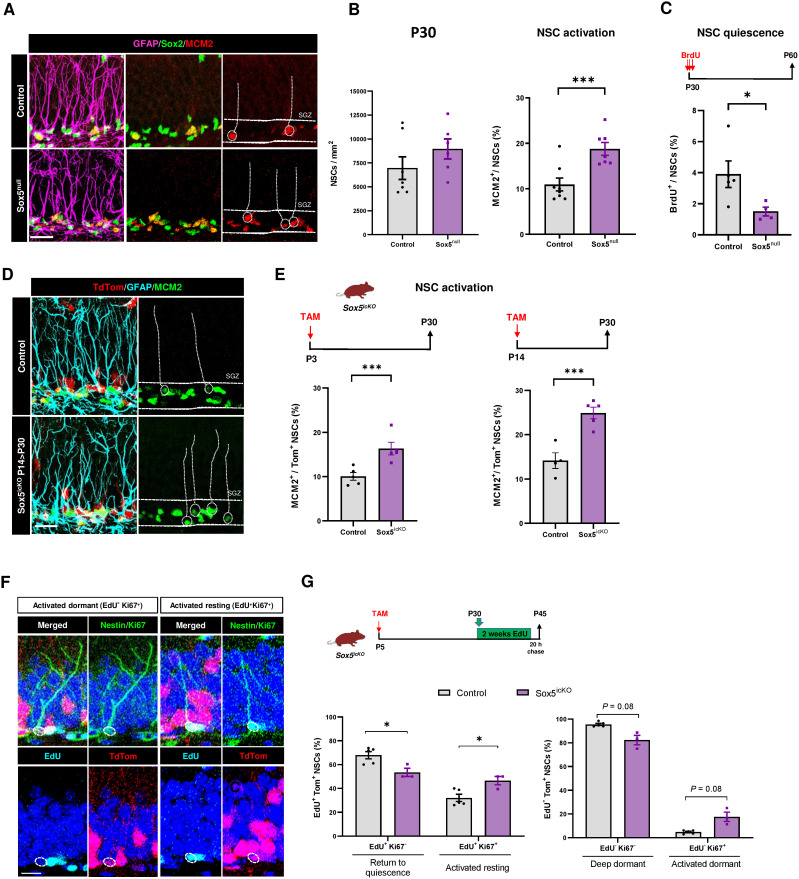
Sox5 loss causes an overactivation and reduced return to quiescence of NSCs in young adult mice. **(A)** Confocal images showing GFAP, Sox2, and MCM2 immunostaining in the SGZ of P30 Control and Sox5^null^ mice. **(B)** Quantitation of NSCs/mm^2^ (rGFAP^+^Sox2^+^ cells), and proliferative MCM2^+^ NSCs (% over total NSCs) of Control and Sox5^null^ P30 mice. **(C)** Scheme of BrdU-long labeling retention experiment. Quantitation of the % of BrdU-L^+^ cells in NSCs (rGFAP^+^Sox2^+^) of P30 Control and Sox5^null^ mice. **(D)** Confocal images showing TdTom, GFAP, and MCM2 immunostaining in the SGZ of P30 Control and Sox5^icKO^ mice. **(E)** Scheme of Sox5 inducible deletion at P3 > P30 and at P14 > P30 in Sox5^icKO^ mice by TAM injection. Quantitation of MCM2^+^ aNSCs in TdTom^+^ rGFAP NSCs of P30 Control and Sox5^icKO^ mice. **(F)** Confocal images showing TdTom expression and Nestin, Ki67, and EdU immunostaining in the SGZ of P30 Control mice after the procedure described in (G). **(G)** Experimental scheme of EdU label retention experiment in P5 > P30 in Sox5^icKO^ mice to label all shallow/resting and aNSCs (EdU^+^) during the P30 > P45 time period. EdU^−^ NSCs could correspond to dormant qNSCs. Quantitation of the indicated subpopulation in the shallow/resting qNSC EdU^+^TdTom^+^ population (left) or in the dormant EdU^-^TdTom^+^ NSC population of P45 Control (*n* = 5) and Sox5^icKO^ (*n* = 3) mice. In all graphs, data represent mean values ± SEM. *p < 0.05, **p < 0.01, and ***p < 0.001 by unpaired Student *t* test. Scale bars represent 50 µm (A and D) and 10 µm in F. Data are available in S1 Data as a part of Suppor*t*ing information. Drawings were created through SciDraw.

If there were an excess of NSCs in a shallow state of quiescence at P14 in Sox5^null^ mice, it could potentially compromise the normal activation/quiescence balance in the young adult neurogenic niche. In fact, in P30 Sox5^null^ mice we observed that the % of aNSCs was surprisingly higher than in Control mice (18.8 ± 1.4 versus 10.9 ± 1.4% of MCM2^+^ cells in the pool of rGFAP^+^ Sox2^+^ cells; *P *= 0.0018; Fig 4A and 4B). Moreover, using Sox5^icko^ mouse to delete Sox5 expression postnatally we confirmed that P30 NSCs showed a higher rate of proliferation in Sox5^icko^ than in Control mice from P3 > P30 (16.3 ± 1.4% versus 10.1 ± 0.9%; *P* = 0.0056; Fig 4D and 4E) or from P14 > P30 (24.9 ± 1.3% versus 14.2 ± 1.7%; *P *= 0.0015; Fig 4D and 4E). These data demonstrate that loss of Sox5, either during development or at different postnatal weeks, leads to the same output in the established young adult DG neurogenic niche: an excess of young adult aNSCs at P30.

Conversely, using BrdU-L retention assays in Sox5^null^ mice from P30 to P60 we observed a clear decrease in the proportion of BrdU-L^+^ NSCs with respect to Control mice (1.5 ± 0.3% versus 3.9 ± 0.8%, respectively; *P* = 0.0465; Fig 4C) indicating that NSCs are more active or that have a lower tendency to enter quiescence in Sox5^null^ mice. These results reinforce the data that young adult NSCs in Sox5^null^ P30 mice are more engaged in activation and less in remaining in quiescence than Control NSCs.

It has been described that aNSCs in young DG return to a resting state of shallow quiescence. These resting cells have a higher activation rate and greater contribution to neurogenesis than dormant cells, which have not left quiescence [10]. To determine if the reduction in qNSCs from P30 to P60 upon Sox5 loss was due to a defect in the return to quiescence of the aNSCs or an activation of dormant qNSCs, we resourced to a saturating EdU retention assay [10]. EdU was administered in drinking water at P30 for two weeks to label proliferating and resting/shallow NSCs, followed by 20-h chase before culling at P45 (Fig 4G). We could identify NSCs in a resting/shallow quiescence as those that have incorporated and retained EdU and the dormant quiescent pool as those NSCs that remained EdU^−^ during the two-week period. Upon Sox5 loss by TAM injection at P5, we observed in P45 Sox5^icKO^ mice an increase in re-activated resting NSCs (46.5 ± 3.5 versus 32.1 ± 2.8% of EdU^+^Ki67^+^ NSCs in the EdU^+^TdTom^+^ NSC pool; *P *= 0.028; Fig 4F and 4G) in comparison with Control mice. However, no significant differences were observed in the dormant EdU^−^ NSC pool, which constitute the majority of the NSC population. In other words, upon Sox5 loss NSCs showed a lower tendency to return to quiescence after proliferating with respect to those in Control mice.

In summary, our results suggest that loss of Sox5 provokes, in the young adult neurogenic niche, an increase in the activation of NSCs and an impaired return to quiescence after proliferation probably due to an excess of NSCs in a shallow state of quiescence.

### Over activation of NSCs provokes a transient increase in neurogenesis in young adult Sox5^null^ mice and a premature depletion of the NSC pool

**Fig 5:**
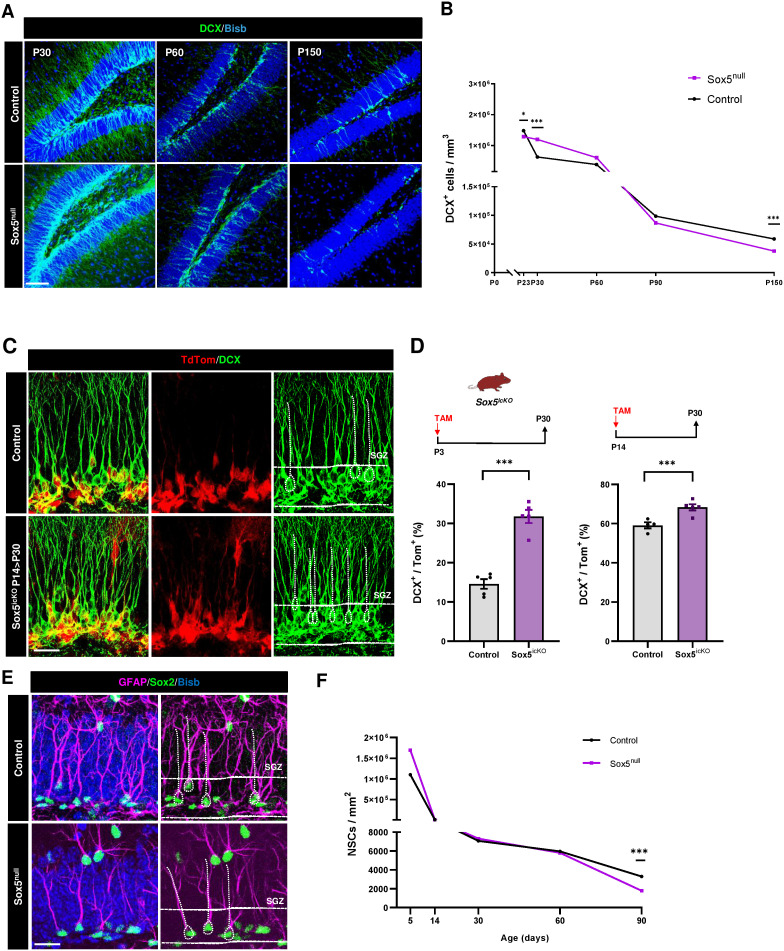
Overactivation of NSCs provokes a transient increase in neurogenesis in young adult Sox5^null^ mice and a premature depletion of the NSC pool. **(A)** Confocal images showing DCX immunostaining in the SGZ of DG at the indicated stages in Control and Sox5^null^ mice. **(B)** Time-course quantitation of DCX^+^ cells/mm^3^ at the indicated stages in Control and Sox5^null^ mice. **(C)** Confocal images showing TdTom^+^ and DCX^+^ immunostaining in the SGZ of P30 Control and Sox5^icKO^ mice. **(D)** Scheme of Sox5 inducible deletion at P3 > P30 and at P14 > P30 in Sox5^icKO^ mice by TAM injection. Quantitation of the % of DCX^+^ cells over total TdTom^+^ cells in P30 Control and Sox5^icKO^ mice. **(E)** Confocal images showing GFAP and Sox2 immunostaining in the SGZ of P90 Control and Sox5^null^ mice. **(F)** Quantitation of NSCs (rGFAP^+^Sox2^+^ cells)/mm^2^ at the indicated stages in Control and Sox5^null^ mice. At least three animals and three sections/animal were analyzed for each immunostaining. In all graphs, data are mean value ± SEM. *p < 0.05, **p < 0.01, and ***p < 0.001 by unpaired Student *t* test. Horizontal lines in pictures indica*t*e the SGZ. Scale bars represent 100 µm (A) and 30 µm (C, E). Data are available in S1 Data as a part of Supporting information. Drawing was created through SciDraw.

In order to determine if the excess in activation and reduced return to quiescence of young adult NSCs in Sox5^null^ mice could have any consequence in neurogenesis, we analyzed the number of DCX^+^ immature neurons. At P14 and P23, a decrease in immature neurons was detected in Sox5^null^ with respect to Control mice (3214 ± 124 versus 4295 ± 289 DCX^+^ BrdU^+^ cells/mm^2^; *P* = 0.04; S2 Fig and 1.3 × 10^6^ ± 7.5 × 10^4^ versus 1.5 × 10^6^ ± 7.0 × 10^4^ cells/mm^3^; *P* = 0.05; Fig 5B). However, by P30 new neurons doubled in Sox5^null^ mice with respect to control mice (1.2 × 10^6^ ± 0.1 × 10^6^ versus 0.6 × 10^6^ ± 0.1 × 10^6^ cells/mm^3^, respectively; *P* = 0.0009; Fig 5A and 5B), indicating that by P30 the excess of aNSC described (Fig 4B) could be responsible for an increase in neurogenesis in Sox5^null^ mice.

To confirm that this defect was not due to embryonic changes in neuronal cell fate decisions in Sox5^null^ mice, we promoted postnatal Sox5 loss at P3 and P14 by TAM-induced recombination in Sox5^icko^. Again, we observed a robust increase in DCX^+^ neurons in the TdTom^+^ recombined cell population both at P3 > P30 and P14 > P30 Sox5^icko^ mice in relation with Control mice (31.8 ± 1.7% versus 14.6 ± 1.2%, *P* < 0.0001 and 68.3 ± 1.6% versus 59.1 ± 1.6%, *P* = 0.005, respectively; Fig 5C and 5D). Thus, loss of Sox5 before the establishment of the adult neurogenic niche (either at embryonic or early postnatal stages) leads to an excess in neuronal production in young adult mice by P30.

Next, we explored if the excess of adult neurogenesis in P30 remained along Sox5^null^ mice life. We did not observe significant changes in the number of DCX^+^ cells at P60 and P90 between Sox5^null^ and Control mice (Fig 5A and 5B), indicating that the excess of neurogenesis at P30 could be compensated later on. Unexpectedly, by P150 the number of new neurons significantly decreased in Sox5^null^ with respect to Control mice (37618 ± 6786 versus 59956 ± 5948 cells/mm^3^, *P* = 0.04; Fig 5A and 5B), indicating that Sox5 is required for long-term neurogenesis.

There are certain situations, such as loss of pro-quiescence genes (Mfge8) [23], epileptogenic conditions [29], or BMP inhibition in aging mice [30], in which NSC over-activation and excess in neuronal production eventually leads to NSC depletion. In fact, although there were not changes in the size of the NSC pool from P14 to P60 between Control and Sox5^null^ mice, by P90 we detected a dramatic depletion of the adult NSC pool in mutant mice with respect to Control (1780 ± 242 versus 3299 ± 180 cells/mm^2^, *P* = 0.0013; Fig 5E and 5F). In summary, these results indicate that NSC over-activation in young P30 Sox5^null^ mice leads to a temporal excess in neuronal production that later derives in a premature depletion of the NSC pool and diminished neurogenesis. Moreover, our results suggests that early control of the right balance of activated and quiescent states is crucial for the long-term maintenance of the adult neurogenic niche.

### Transcriptomic analysis of Sox5^null^ NSCs reveals dramatic changes in developmental programs in the adult neurogenic niche, including BMP canonical pathway activation

**Fig 6:**
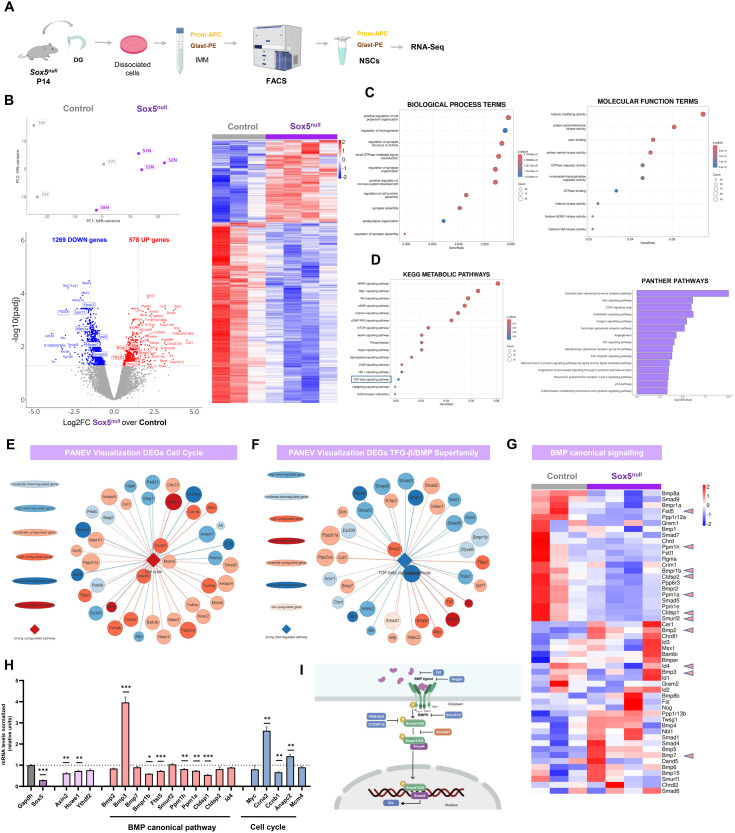
Transcriptomic analysis of Sox5^null^ NSCs at P14 reveals important changes in developmental programs, including activation of cell cycle and canonical BMP signaling pathway. **(A)** Experimental approach of RNA-Seq analysis of FACS-sorted NSCs from Control and Sox5^null^ P14 mice. **(B)** Principal Component Analysis (PCA) of DEGs indicating independent segregation of the two genotypes analyzed, Control (gray) and Sox5^null^ (violet). Heat Map of DEGs in Sox5^null^ vs. Control. Volcano Plot of Log2FC and −log10 (padjusted) of DEGs between Control and Sox5^null^ mice. Several relevant genes are indicated in boxes, and those related to quiescence (*Foxo3, Foxo4, Huwe1*) are downregulated, whereas genes related to cell cycle (*Myc*) and BMP canonical signaling (*BMP2, BMP4, BMP5*) are upregulated in Sox5^null^ vs. Control DG. **(C)** Enrichment analysis of GO terms related to Biological Processes and Molecular Functions in DEGs of Sox5^null^ vs. Control NSCs. **(D)** KEGG and PANTHER pathways analysis of DEGs of Sox5^null^ vs. Control NSCs. DEGs were selected for their *p*-adjusted value < 0.05. **(E, F)** Node graphs of most representative upregulated cell cycle genes and downregulated TGF-β/BMP Superfamily signaling pathway genes generated by the PANEV software (PAthway NEtwork Visualizer; v. 17.0). **(G)** Heat Map of DEGs manually curated in the list of BMP signaling pathway genes comparing Sox5^null^ vs. Control NSCs. **(H)** Quantitation of the relative levels of mRNA expression by quantitative PCR for the indicated transcripts in Sox5^null^ vs. Control NSCs prepared in vitro (see Fig 7). Results are shown as 2^−ΔΔCT^ normalized with respect to *GAPDH* mRNA and relative to P14 NSCs Control values (dashed line y-axis = 1). Control and Sox5^null^ P14 mice (*N* = 3 and 3, respectively) were used to prepared DG NSC cultures and were analyzed for each condition and experimental replicates were performed. **(I)** Scheme of the BMP canonical signaling pathway. Data represent mean values ± SEM. *p < 0.05; **p < 0.01; ***p < 0.001according to a Student *t* test. Data are available in S1 Data as a part of Supporting information. Drawings were created through SciDraw, Bioicons, Bioart, and Inkscape.

To elucidate the molecular mechanisms behind the profound alterations in NSC activation and quiescence observed upon Sox5 loss, we performed RNA sequencing (RNA-Seq) comparative analysis in Control and Sox5^null^ NSCs (Fig 6A). First, NSCs from P14 DG were acutely isolated by FACS sorting using the combination of cell surface markers GLAST and Prominin-1 [7] (S3 Fig). Then, we confirmed by RT-qPCR and by RNA-Seq that the isolated Prom^+^ GLAST^+^ enriched cells in the DG of P14 mice had NSC transcriptional profile (S3E–S3F Fig) [31] although there were probably some astrocytes in the FACS-sorted pool.

We then performed a RNA-Seq analysis of Control and Sox5^null^ enriched NSCs at stage P14 (*n* = 3 and 4, respectively; Fig 6). We first established by principal component analysis of differentially expressed genes (DEGs; adjusted *p*-value < 0.05; Deseq^2^ software) that our individual samples segregated clearly into Control and Sox5^null^ groups (Fig 6B). Similarly, the distribution in a volcano plot showed 1269 genes downregulated and 578 genes upregulated (Log2Fold Change, Log2FC> +0.5) in the NSCs of Sox5^null^ mice with respect to Control ones (Fig 6B). Furthermore, the heat map of DEGs clearly showed individual differences in the level of expression of 1847 genes between groups (Fig 6B). To capture biological information on DEGs, Gene Ontology (GO) analysis for biological processes highlighted changes in development (regulation of neurogenesis, positive regulation of nervous system development) and in neuronal cell fate (synapse structure or activity, synapse organization; Fig 6C). These DEGs could be at the molecular base of the changes in NSC proliferation and quiescence previously described (Figs 4 and 5). Interestingly, GO analysis for molecular functions highlighted changes in signaling pathways involving serine-threonine kinases, GTPases, and histone modifications that point to important signaling pathways alterations in Sox5^null^ NSCs (Fig 6C).

Using KEGG Software to explore those alterations in signaling pathways upon Sox5 loss, we found several clusters affected, including MAPK kinases and Wnt signaling associated with NSC proliferation [32,33] (Fig 6D). Moreover, we found TGF-β superfamily signaling in the KEGG list, a pathway that has been strongly associated to NSC quiescence regulation [15,34] (Fig 6D). In order to determine if the combination of DEGs were indicative of up or downregulation of each of the pathways they were associated to, we resourced to PANEV (PAthway NEtwork Visualizer) [35] a recently described R package set for gene/pathway-based network visualization based on information available on KEGG. We observed a high upregulation of the cell cycle, especially with upregulated genes such as *Myc*, *Mcm4*, cyclins *CcnA2* and *CcnB1*, and *Anaphase Promoting Complex Subunit 2* (*Anapc2*; Fig 6E). These data were further confirmed by qPCR in P14 Control and Sox5^null^ mice NSCs grown in culture, showing a clear increase in several cell cycle mRNAs in Sox5^null^ P14 NSCs in vitro with respect to Control mice (Fig 6H). We also confirmed the downregulation of *Axin2* a direct target of Sox5 (Fig 6H). Although, NSCs from Sox5^null^ P14 mice showed a lower proliferation rate (lower number of NSCs expressing Mcm2 protein; Fig 3B and downregulation of Wnt signaling pathway, S4A Fig), they exhibit a reduced maintenance of the quiescence state (Fig 3D). Thus, the overall increase in cell cycle activation at the mRNA level (Fig 6E and 6H) in a population of NSC with low proliferation rate (Fig 3B) could be indicative of NSCs in a primed-like state prepared for cell cycle entry [8].

PANEV analysis also predicted a strong downregulation of the TGF-β/BMP Superfamily signaling pathway (Fig 6F). However, the TGF-β Superfamily is a large and diverse group of structurally related secreted growth factors. Whereas members of the TGF‐β/activin/nodal class signal through TGFRs to activate Smad2/3 mediated signals, members of the BMP class signal mostly through BMPRs to activate Smad1/5/9 proteins and downstream targets such as ID proteins (Fig 6I). In a selection of DEGs in Sox5^null^ NSCs ascribed to the BMP canonical signaling pathway, we observed several upregulated BMP ligands such as *Bmp2, Bmp3, Bmp7*. However, BMP ligands inhibitors (*Smurf* and *Flst5*) and phosphatases responsible for the shutdown of BMP canonical signaling through dephosphorylation of pSmad1/5/9 (*Ppm1a*, *Ppm1e*, *Ppm1h*, *Ctdsp1*, and *Ctdsp2*; Fig 6I) [36,37] were clearly downregulated (Fig 6G). Several of these changes in BMP canonical signaling pathway were then confirmed by qPCR in P14 Control and Sox5^null^ mice NSCs grown in culture (Fig 6H). These data suggest that BMP canonical pathway could be upregulated upon Sox5 loss in P14 NSCs.

In summary, bulk transcriptomic analyses in enriched NSC population revealed that Sox5 loss of expression causes marked changes in developmental programmes during P14, including an increase in cell cycle transcripts in a population suggestive of a primed state and upregulation of the BMP canonical signaling pathway.

### Sox5 is required to prevent an excess of BMP canonical signaling activation in NSCs in vitro

**Fig 7:**
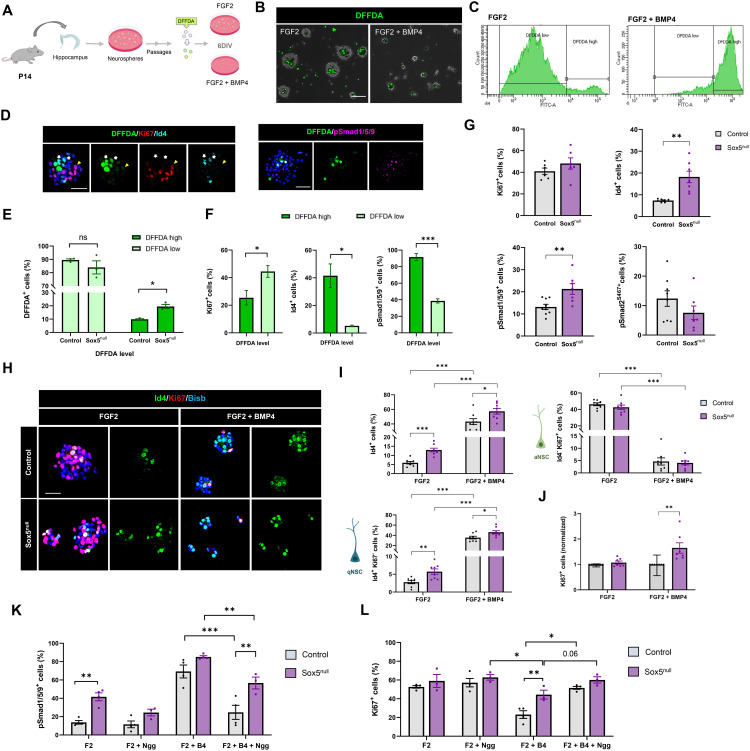
Sox5 is required to prevent an excess of BMP signaling pathway in NSCs during the second postnatal week in vitro. **(A)** Methodological approach for NSC culture. **(B)** Representative bright field images of P14 neurospheres loaded with DFFDA (green) under proliferating (FGF2) and quiescence (FGF2 + BMP4) culture conditions for 6 days. **(C)** Quantitation using flow cytometry of DFFDA^high^ and DFFDA^low^ cells at the indicated culture condition. **(D)** Confocal images showing DFFDA fluorescence and Ki67, Id4, or pSmad1/5/9 expression in P14 NSCs. **(E)** Quantitation of DFFDA^high^ and DFFDA^low^ cells in Control and Sox5^null^ mice in proliferation. **(F)** Quantitation of the percentage of pSmad1/5/9^+^, Ki67^+^, and Id4^+^ cells among DFFDA^high^ and DFFDA^low^ cells from Control mice cultured in FGF2. **(G)** Quantitation of the percentage of pSmad1/5/9^+^, Ki67^+^, and Id4^+^ cells relative to total NSCs in Control and Sox5^null^ mice cultured in FGF2 (passages 2–6). **(H)** Confocal images showing Ki67 and Id4 immunostaining in the indicated conditions. **(I)** Quantitation of the percentage of Id4^+^ cells, Id4^+^/Ki67^−^ (qNSCs), and Id4^−^/Ki67^+^ (aNSCs). **(J)** Quantitation of total Ki67^+^ cells relative to total cell number in Control and Sox5^null^ mice under the indicated culture conditions. **(K, L)** Quantitation of the percentage of pSmad1/5/9^+^ (K) or Ki67^+^ (L) cells relative to total NSCs in Control and Sox5^null^ mice cultured in FGF2 or FGF2 + BMP4, with or without the addition of the BMP signaling inhibitor Noggin (Nog), as indicated. Three independent experiments were performed of control and Sox5^null^ P14 DG NSCs (2–3 different animal of each). At least 500 cells were analyzed for each condition. Data represent mean values ± SEM. *p < 0.05; **p < 0.01; ***p < 0.001 according to a Student *t* test (E–G) and two-way ANOVA test (I–M). Scale bars represent 100 µm (B) and 30 µm (D, H). Data are available in S1 Data and S2 Data as a part of Supporting information. Drawings were created through SciDraw, Bioicons and Inkscape.

To further explore if possible changes in BMP canonical pathway could be behind those alterations observed in P14 Sox5^null^ NSCs, we resourced to an in vitro model in which hippocampus-derived NSCs were grown as floating neurospheres in the presence of mitogens (Fig 7A) [26]. To capture activated and quiescent states, P14 hippocampal NSCs expanded by several passages were loaded with a cell tracer (DFFDA-Oregon Green fluorophore) that is diluted over cell divisions (Fig 7A) [12]. In this culture, dormant quiescent NSCs neither engage in cell cycle nor survive past 48 h [12,38], whereas cells in a primed state retain high levels of DFDDA and contribute predominantly to the long-term maintenance of the NSC culture [12]. After six days in culture in the presence of FGF2, we determined by FAC that the majority of P14 NSCs diluted the cell-tracer upon cell division (79.8 ± 7.2%, DFFDA^low^) whereas few cells retained high DFDDA levels (19.6 ± 7.2%, DFFDA^high^; Fig 7B and 7C). Moreover, adding BMP4 that promotes quiescence entry [15] in combination with FGF2, we observed a distinct shift in the cell profile as 80.4 ± 5.3% of cells were DFFDA^high^ and only 19.3 ± 5.3% were DFFDA^low^ (Fig 7B and 7C). We then compared Sox5^null^ and Control P14 NSCs and observed a significant increase in the % of DFFDA^high^ cells in Sox5^null^ NSCs grown in FGF2 compared to Control (20.0 ± 2.5% versus 9.9 ± 0.4%; *P* = 0.007; Fig 7E). These results indicate that Sox5 loss promotes the generation of a higher number of primed-like qNSCs identified as DFFDA^high^ cells.

As BMP canonical pathway has been associated to the quiescent state [15,39] and we had observed several upregulated DEGs of that pathway in P14 Sox5^null^ NSCs (Fig 6G and 6H), we tested several BMP canonical targets that could be associated with the primed-like state of NSCs in vitro. Thus, we found that Id4 (a BMP signaling effector) [14,15] and phosphorylated Smad1/5/9 (pSmad1/5/9, a mediator and direct indicator of BMP canonical signaling activation), were enriched in P14 DFFDA^high^ cells with respect to DFFDA^low^ cells (41.5 ± 8.6% versus 5.2 ± 0.5%; *P* = 0.05 and 91.5 ± 4.3% versus 38.6 ± 2.5%; *P* = 0.0005, respectively; Fig 7D and 7F). By contrast, the opposite distribution was observed for cell cycle marker Ki67 (25.4 ± 5.2% versus 44.5 ± 4.2%; *P* = 0.047; Fig 7D and 7E). These results indicate that NSCs DFFDA^high^ in vitro are characterized by high levels of BMP canonical pathway activation.

We next checked if those BMP canonical signaling indicators could be altered in acute Sox5^null^ NSCs in culture, from passage 0 (primary) to passage 6. We did not observe significant changes in the number, size, and proliferation (Ki67^+^ cells) of Sox5^null^ NSCs with respect to those of Control mice (S5 Fig and Fig 7G). However, we observed a consistent upregulation of BMP canonical pathway mediator pSmad1/5/9 and effector Id4 in Sox5^null^ NSCs with respect to Control NSCs (21.3 ± 2.4% versus 13.2 ± 1.2%; *P* = 0.006 and 18.2 ± 2.6% versus 7.4 ± 0.3%; *P* = 0.006, respectively; Fig 7G), whereas TGF-β/Activin canonical pathway mediator pSmad2 was not significantly affected (Fig 7G). Thus, Sox5 is required to supress BMP canonical pathway in postnatal NSCs.

After expanding control and mutant NSCs for several passages (5–15 passages), we explored Sox5^null^ NSCs responsiveness to a canonical BMP pathway ligand such as BMP4. We observed a similar response in Control and Sox5^null^ NSCs, as the number of Id4^+^ cells dramatically increased when NSCs were exposed to BMP4 + FGF2 with respect to FGF2 condition (43.3 ± 3.9% versus 6.0 ± 0.7%; *P *< 0.0001 and 57.3 ± 3.8% versus 13.0 ± 1.0%; *P *< 0.0001, respectively Fig 7H and 7I). However, Id4^+^ NSC number was higher in Sox5^null^ with respect to Control NSCs in both conditions, reinforcing the idea that Sox5 prevents an excess of BMP canonical activation both in proliferating and quiescence conditions.

Moreover, when classifying Id4^+^ NSCs according to Ki67 expression, we did not observe significant differences in aNSCs (Id4^−^/Ki67^+^). However, there were a significant increase in the % of Id4^+^/Ki67^−^ quiescent cells in Sox5^null^ in comparison with control NSCs in both conditions (FGF2: 5.7 ± 0.7% versus 2.8 ± 0.4%; *P* = 0.004; FGF2 + BMP4: 46.1 ± 3.1% versus 35.5 ± 2.7%; *P* = 0.02; Fig 7H and 7I). These results stress the point that Sox5 prevents an excess of quiescent NSCs as observed in Fig 7E using DFFDA retention assay. However, we also observed that independently of Id4 expression, Sox5^null^ NSCs maintained higher rate of proliferation when forced to enter quiescence by BMP4 (1.8 folds ± 0.21% versus 1.0 ± 0.27%; *P* = 0.0084; relative normalized levels with respect to Control; Fig 7J), In summary, these results suggest that Sox5^null^ NSCs have alterations in the response to BMP canonical signaling, showing abnormally high levels of pSmad1/5/9 and Id4 and hindered quiescence entry in vitro.

To determine if this excess in BMP canonical signaling was responsible for the defects in quiescence depth, we used a potent inhibitor of BMP ligand binding, Noggin (Nog), to try to rescue the defects in Sox5^null^ NSCs. First, in basal FGF2 condition, Nog prevented the increase in pSmad1/5/9^+^ cells in Sox5^null^ NSCs and the levels were similar to those in Control NSCs (24.47 ± 3.7% versus 11.59 ± 3.7%; *P* = 0.08; Fig 7K). Then in the FGF2 + BMP4 condition to force quiescence entry, Nog prevented the increase in pSmad1/5/9^+^ cells in Control NSCs (F2 + B4 + Nog: 24.74 ± 7.68% versus F2 + B4: 69.25 ± 7.04%; *P* = 0.005; Figs 7K and S6A and S6B) and in Sox5^null^, (F2 + B4 + Nog: 65.25 ± 9.71% versus F2 + B4: 85.14 ± 1.32%; *P* = 0.036; Fig 7K). Moreover, proliferation levels measured by Ki67 were similar between Control and Sox5^null^ NSCs when Nog was added in the FG2 + BMP4 condition (51.07 ± 1.55% versus 60.07 ± 3.37%; *P = *0.06; Fig 7L). These results reinforce the idea that Sox5^null^ NSC defects in quiescence depth are in part due to an excess in canonical BMP signaling. However, as pSmad1/5/9+ and Ki67+ cells in Sox5^null^ NSCs are higher with respect to those of Control NSCs even in the presence of Nog, other BMP pathway elements could be acting downstream of the phosphorylation of Smad1/5/9 in Sox5^null^ NSCs.

In summary, these results suggest that loss of Sox5 at P14 provokes an increase in NSCs in a primed-like state that can be identified in vitro by cell tracer retention. Moreover, that state could be caused by the dysregulation of pSmad1/5/9 levels in the Sox5^null^ NSCs, as BMP inhibition partially rescue the proliferation defects.

### Canonical BMP pathway inhibition partially rescue the excess of aNSC in Sox5^null^ young adult DG in vivo

**Fig 8:**
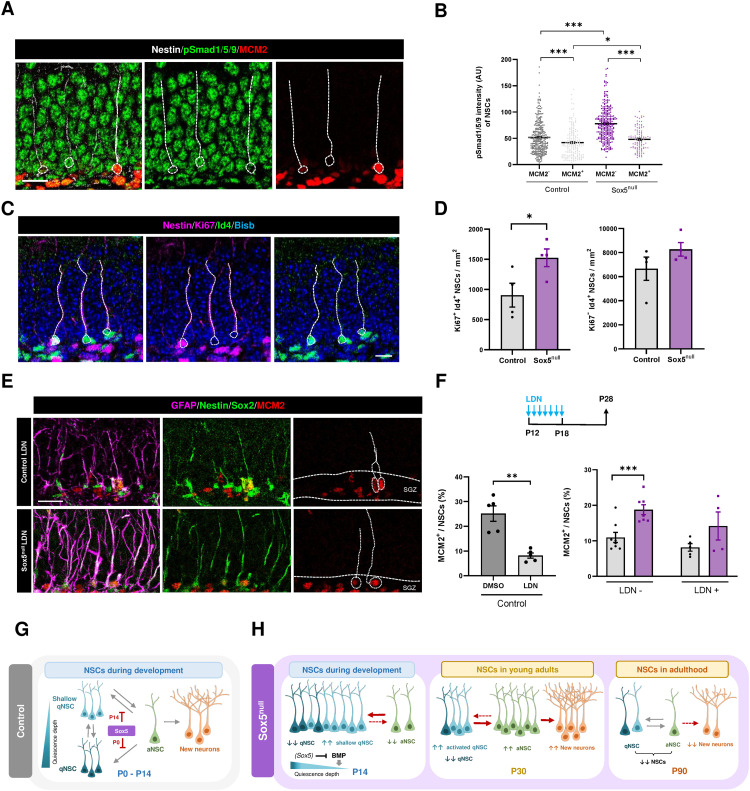
Canonical BMP pathway inhibition partially rescues the excess of aNSCs in Sox5^null^ young adult DG in vivo. **(A)** Confocal images showing Nestin, pSmad1/5/9 and MCM2 immunostaining in the SGZ of P14 Control and Sox5^null^ mice. **(B)** Quantitation of pSmad1/5/9 levels intensity (arbitrary units) in aNSCs (MCM2^+^) and qNSCs (MCM2^−^) in Control and Sox5^null^ P14 mice. **(C)** Confocal images showing Nestin, Ki67, and Id4 immunostaining in the SGZ of P14 Control and Sox5^null^ mice. **(D)** Quantitation of Id4^+^Ki67^+^ and Id4 ^+^ Ki67^−^ Nestin^+^ NSCs per mm^2^ in Control and Sox5^null^ P14 mice. **(E)** Confocal images showing GFAP, Nestin, Sox2, and MCM2 immunostaining in the SGZ of P28 Control and Sox5^null^ mice injected with LDN193189 (LDN). **(F)** Scheme of BMP signaling inhibitor LDN193189 i.p. administration from P12 > P18 in Control and Sox5^null^ mice. Quantitation of MCM2^+^ cells in NSCs (Nestin^+^GFAP^+^Sox2^+^) in Control and Sox5^null^ P28 mice injected with LDN or DMSO. Panel left of the right graph is the same as shown in Fig 4B. **(G, H)** Summary of NSC quiescence dynamics during early postnatal DG development (G) and their alterations in Sox5^null^ mice (H). Data are available in S1 Data as a part of Supporting information. Drawings were created through Inkscape.

To determine if BMP signaling was altered at the protein level in Sox5^null^ NSCs in vivo, we analyzed BMP signaling components in DG sections at P14. We observed that pSmad1/5/9 levels were higher in Sox5^null^ NSCs with respect to those in Control NSCs, both in MCM2^−^ qNSCs (77.79 ± 1.883 versus 51.54 ± 1.738 AU; *P *< 0.0001) and in MCM2^+^ aNSCs (41.93 ± 2.10 versus 48.12 ± 2.09 AU; *P *= 0.037; Fig 8A and 8B). Moreover, we analyzed the expression of the BMP transcriptional target Id4 in P14 Nestin^+^ NSCs. As expected, in Control mice the majority of Id4^+^ cells were Ki67^−^Nestin^+^ qNSCs, whereas aNSCs could be identified as Nestin^+^Ki67^+^Id4^−^ cells. However, we observed few Nestin^+^ NSCs that were double positive (Ki67^+^Id4^+^) and that could represent an intermediate state between quiescence and activation. Surprisingly, Sox5^null^ mice showed an increase in these Nestin^+^ Ki67^+^ Id4^+^ NSCs in comparison with Control mice (1525 ± 146.6 versus 906 ± 196.6 cells/mm^2^; *P *= 0.0452; Fig 8C and 8D). Thus, these data suggest that around P14 in vivo, there is a subpopulation of Id4^+^ NSCs, probably in an intermediate state between activation and quiescence, which is selectively increased by Sox5 loss.

Finally, to explore if the elevated levels of BMP canonical signaling at P14 were behind the excess of activation of NSCs by P30 in Sox5^null^ mice, we resourced to a BMP canonical inhibitor that specifically inhibit BMPR1b phosphorylation and consequently Smad1/5/9 activation [LDN193189 (LDN); S6 Fig] [40]. We observed that daily LDN intraperitoneal injection in Control mice during the critical period around P14 (P12–P18) provoked a clear decrease in NSCs proliferation by P30, in comparison with DMSO injected Control mice. (8.15 ± 1.06 versus 25.14 ± 3.10% of MCM2^+^ NSCs; *P *= 0.0037). These data suggest that BMP signaling during the P14 critical window is required for the correct activation of young adult NSCs by P30.

Moreover, reduction in BMP signaling levels by LDN injection in Sox5^null^ mice from P12 to P18 partially rescued the excess of NSC proliferation previously observed in P30 Sox5^null^ mice (Fig 4B copied now in Fig 7F; LDN^−^) as there were not significant differences with respect to Control LDN injected mice (14.17 ± 3.95 versus 8.15 ± 1.06% of MCM2^+^ NSCs; *P *= 0.23; LDN + ; Fig 7F). These results, suggest that dysregulated high levels of BMP canonical pathway are in part responsible for the aberrant NSC activation observed upon Sox5 loss.

In summary, our data demonstrate that maintaining restricted levels of BMP canonical signaling during postnatal DG development are essential to establish the correct number of qNSCs in the adult neurogenic niche that ensures long-lasting maintenance of the NSC pool.

## Discussion

To understand the process of adult neurogenesis is essential to determine how adult quiescent NSCs emerge during development and how they acquire a reversible state of quiescence. In this study, we have shown that Sox5 transcription factor: (i) restricts NSC quiescence entry during the first postnatal week; (ii) limits the establishment of a shallow/resting quiescent state during the second postnatal week, and (iii) restricts the levels of BMP canonical signaling pathway during DG postnatal development. Moreover, we have established that the defect in the quiescence depth in NSCs leads to a hyper-neurogenic adult NSC pool that is prematurely exhausted. Thus, establishing the correct balance between dormant and shallow/resting quiescent sub-states during postnatal NSC development is essential for long-term maintenance of the neurogenic niche (Summary in Fig 8G and 8H).

The first postnatal week of DG development is critical for the generation of adult NSCs. First, specific elimination of dividing NSCs between P0 and P7, but not during P14–P21, severely impairs the size of the adult NSC pool [41]. Moreover, label-retaining birth-dating experiments revealed that NSC first entry into quiescence occurs during the first postnatal week [1,3] and that Sufu embryonic deletion (which decreases Shh signaling) promote premature quiescence entry during that week [3]. More recently, it has been described that m^6^A/YTHDF2-mediated mRNA decay targets TGF-β signaling to suppress NSC quiescence entry during the first postnatal week [19]. Using long-term retention of thymidine analogues, we now show that Sox5 is a new player in that early process, as it is required during the first postnatal week to prevent an excessive entry of NSCs into quiescence and to promote NSC proliferation. However, whereas Shh reduction or TGF-β increase leads to a maintained reduction in neurogenesis along time [3,19], we have observed that Sox5 loss has revealed another important quiescence modulation during the second postnatal week, when Sox5 is required to prevent an excess of NSCs in a shallow quiescent state as we describe below.

There is an emerging notion in the field that quiescent adult NSCs are at different levels of quiescence depth, forming a continuum of sub-states along the quiescence-activation trajectory. Single-cell and bulk transcriptomic analysis from adult SVZ and SGZ have revealed adult NSCs in intermediate states which includes a shallower or primed state [8,9,12,13,42], cell cycle-low activated state [43] or “resting” state [10]. However, it is unclear when do those intermediate states emerge during the neurogenic niche development. Our work indicates that NSCs acquired a shallow quiescence state along the second postnatal week. Precisely, upon Sox5 loss, both NSC activation and maintenance in quiescence decreased around P14 in vivo. This Sox5^null^ NSC population does not retain BrdU-L for weeks (they are not dormant qNSCs), show a higher rate of co-expression of quiescence marker Id4 and proliferating marker Ki67 and exhibit a transcriptional profile of cell cycle upregulated genes. All these data would suggest that quiescent NSCs at P14 in Sox5^null^ are in a shallow/primed state of quiescence. We have reinforced this idea by in vitro DFFDA cell tracer retention assays [12], showing that the pool of NSCs are in a primed-like state (DFFDA^high^) and is enlarged in P14 Sox5^null^ mice. Moreover, upon forcing Sox5^null^ NSCs to enter in deep quiescence (BMP4 addition in culture) they are maintained in proliferation at higher rates than Control NSCs. Consequently, we propose that around P14 there is a critical window to establish the correct deepness of quiescence in the hippocampal NSCs and that it is essential to restrict the number of shallow quiescent NSCs. Moreover, as NSCs can only undergo a limited number of rounds of cell division prior to terminal differentiation [44], that early decision of preventing an excess of NSCs in a shallow quiescence is fundamental to establish a long-lasting NSC pool and neurogenic capacity in adult mice.

Our results are reinforced by recent studies indicating that autophagy is required for the acquisition and maintenance of NSC quiescence in the first postnatal weeks [7,11] and by CcnD2 mutant analysis, which suggest that P14 NSCs are in a transition from developmental to adult NSC state [45]. The absence of CcnD2 does not affect normal development of the DG until birth, but prevents postnatal formation of adult NSCs which are born on-site from precursors located in the DG shortly after birth. Our analysis would indicate that the developmental to adult NSC transition could include the establishment of a shallow quiescence state controlled in part by Sox5 activity.

Recent studies have described that the probability of adult NSCs to transit to a shallow/resting quiescence state increases over time, comparing one-month old and aging DGs [10]. Ascl1 plays a relevant role in the maintenance of that resting state in adult NSCs as in *Ascl1* hypomorph mice there is an increase in the proportion of adult NSCs that return to quiescence after cell division and, consequently, an enlarged NSC pool [4,10]. Opposite to what has been described in Ascl1 deficient mice, in young Sox5^null^ adult mice pNSCs return less to quiescence after cell division and NSC activation increases. This excess of resting qNSC activation could be sustained by deep transcriptomic changes, including upregulation of cell cycle activators and MAPK pathway, as we have described. Moreover, those alterations cause a progressive reduction of the NSC pool and have a deleterious effect in elder Sox5^null^ mice neurogenesis. While Sox5 and Ascl1 may exhibit contrasting roles in regulating the return of resting qNSCs to dormant quiescence in young adult mice, it remains unclear whether Ascl1 participates in the initial establishment of a primed state in developmental NSCs, akin to the role described here for Sox5.

Regarding possible mechanisms controlling the shallow quiescence state, we have found that BMP canonical signaling pathway through pSmad1/5/9 and Id4 expression could play a fundamental role in promoting a shallower quiescence state in NSCs during the first postnatal weeks (Fig 8H). BMP canonical pathway controls several aspects of DG development and function. Thus, during development, inhibition of embryonic BMP canonical signaling in NSCs impairs DG neurogenesis [46]. Furthermore, BMP canonical pathway components such as BMP4 and its downstream effectors Id1 and Id4 are essential for quiescence maintenance in adult NSCs [14–18]. Now, we have described that Id4 expression at P14 could be indicative of an intermediate/shallow state in NSCs when combined with Ki67 expression, both in vitro and in vivo. Importantly, we have shown that blocking BMP canonical activity during the P14 window has a strong impact on the activation of young adult NSCs and partially rescues the defects in Sox5^null^ NSCs, both in vivo and in vitro. Moreover, Sox5 is crucial to prevent an excess of BMP canonical pathway activation in NSCs as indicated by our in vivo immunostaining of pSmad1/5/9, bulk RNA-Seq, RT-qPCR analysis, and in vitro NSC immunostaining (Id4 and pSmad1/5/9).

Our work also points to the fact that developmental loss of Sox5 could have severe consequences on hippocampal function, as new DG neurons promote flexible learning and adaptive behavioral and endocrine responses to cognitive and emotional challenges. Moreover, given the fact that *SOX5* heterozygous inactivating variants cause neurodevelopmental disorders in humans (Lamb-Shaffer Syndrome; OMIM # 616803), our findings will also help to understand the episodic and social memory deficits described in those patients [47].

## Materials and methods

### Mice

All experiments were performed in postnatal three day-old (P3) to 5-months-old C57BL/6J background mice of both genders. Animal procedures were carried out in accordance with the guidelines of European Union (2010/63/UE), Spanish legislation (53/2013, BOE no. 1337) and under the approved projects (PROEX 078/17 and PROEX 146.0/22). Mice were housed with a standard control of a 12 h light/dark cycle and maintained in the animal facility at Cajal Institute.

Sox5^fl/fl^ mice, in which coding exon 5 of Sox5 gene is flanked by loxP sites were used [28]. For conditional mice, they were bred with Nestin-Cre mice (RRID:IMSR_JAX:003771) to generate control animals (Control): Nestin-Cre/Sox5^fl/+,^ Sox5^fl/fl^ or Sox5^fl/+^ and mutant mice: Nestin-Cre/Sox5^fl/fl^ (Sox5^null^) For conditional inducible mice, they were bred with Sox2-creER^T2^ mice [48] and with Rosa26-loxP-stop-LoxP-TdTomato (RRID: IMSR_JAX:007908) reporter mice to generate control animals (Control): Sox2-creER^T2^/TdTom^+^ or Sox2-creER^T2^/Sox5^fl/+^/TdTom^+^ and mutant mice: Sox2-creER^T2^/Sox5^fl/fl^/TdTom^+^ (Sox5^icKO^).

### Tamoxifen treatment and administration of BrdU/EdU and LDN193189

For activation of the CreER^T2^ protein, 5 mg/40 g body weight of tamoxifen (TAM, 10 mg/ml in corn oil; Sigma) was intraperitoneally (i.p.) injected once (in P3 mice) or three times, one injection every 24 h (in P14 mice).

The labeling of cells progressing though S-phase was performed by either i.p. injections of BrdU (Roche) or EdU (Sigma Aldrich) (50 mg/kg) using PBS as vehicle or through administration of EdU in the drinking water (0.2 mg/ml). Animals were injected once (P3 and P14 mice) or five times with one daily injection (1-month-old mice) and sacrificed 15 or 30 days after. For long-term (14 days) EdU administration in water, fresh solution was replaced at least every 56 h.

For the inhibition of the BMP pathway, a selective BMP receptor antagonist, LDN193189 (LDN, Sigma-Aldrich), was injected i.p. once a day for 7 days from P12 to P18 at 3 mg/kg per mice. LDN was thoroughly diluted in DMSO (10 mg/ml) and freshly prepared in a 1:39 ratio with saline solution (NaCl 9 mg/ml) before administration. LDN193189 is a derivative of dorsomorphin that is a highly selective antagonist of BMP receptor isotypes ALK2 and ALK3 (IC50 of: 5 and 30 nM). The selectivity of LDN193189 for ALK2/3 is 200 fold over the TGF-β type receptors ALK4, −5, and −7.

### Tissue preparation and immunofluorescence

Animals were transcardially perfused with saline followed by 4% paraformaldehyde (PFA). Brains were postfixed with 4% PFA for 3 h at 4 °C, embedded in 30% agarose/sucrose (w/v), and coronally sectioned in 50 μm slices using a vibratome.

For immunostaining, vibratome floating brain sections were permeabilized with 1% Triton X-100 in 0.1M Phosphate Buffer (PB) for 30 min and blocked with 10% Fetal Bovine Serum (FBS) and 0.25% Triton X-100 in 0.1M PB for 2 h at room temperature with rocking. For fixed cells, permeabilization and blocking were performed with 10% FBS in 0.25% Triton X-100 in 0.1M PB for 1 h at room temperature. Primary antibodies: BrdU (1:500, ab6326 Abcam), DCX (1:500, ab18723 Abcam), GFAP (1:1000, Z0334 Dako), Hopx (1:500, sc-398703 Biogen), pSmad1/5/9 (1:500, ab92698 Abcam), pSmad2^S467^(1:500, ab280888 Abcam) Id4 (1:500, BCH-9/#82-12 BioCheck), Ki67 (1:500, 550609 Bd Biosciences), MCM2 (1:1000, 610701 Bd Biosciences), Prox1 (1:1000, MAB5654, Millipore), Sox5 (1:500, A. Morales), Sox2 (1:500, AF2018 R&D), Tbr2 (1:1000, ab216870 Abcam), were prepared in incubation buffer (1% FBS, 0.25% Triton X-100 in 0.1M PB) and incubated with sections or cells overnight at 4 °C. Following three washes with washing buffer (0.1% Triton X-100 in 0.1M PB), immunoreactivity was detected using appropriate Alexa Fluor-conjugated (Life Technologies, Invitrogen and Abcam) secondary antibody (1:1000) diluted in incubation buffer for 2 h at room temperature. After three washes, sections or cells were incubated with bisbenzimide (1:100 in 0.1M PB) for 2 min at room temperature and mounted using Fluoromount-G (Thermo Fisher).

To detect MCM2 protein, antigen retrieval was carried out by incubating sections in 0.15M sodium citrate at 80 °C for 30 min. To detect BrdU incorporation, DNA was denatured by incubating sections or cells with 2N HCL for 25 min at room temperature, followed by 0.15M boric acid neutralization for 20 min. To detect EdU signal was used “EdU IV Imaging Kit 647 S” based on the Click-it reaction with azides conjugated to fluorochromes (Sigma Aldrich, BCK647-IV-IM-S) following instructions of the manufacturer.

### FACS

For hippocampal NSC isolation, DGs of Control and Sox5^null^ P14 mice were dissected and pooled (two animals per sample; *N* = 3 and 4, respectively). Tissue was dissociated using Papain **[**1.5 mg/ml papain (Worthington) + 0.2 mg/ml cysteine (Sigma) + 0.2 mg/ml EDTA (Merck) + Hank´s buffer (Thermo Fisher)] for 20 min at 37 °C. After mechanical dissociation, cell suspension was filtered (70 µm, Miltenyi) and labeled with GLAST(ACSA-1)-PE (1:40, Miltenyi, 130-118-483) and Prominin-1(MB9-3G8)-APC (1:20, Miltenyi, 130-123-793) for 15 min at 4 °C. NSCs were isolated by FACS (BD FACSaria Fusion Cell Sorter) using an 85 µm nozzle. Compensations were done on single-color cell controls and gates were set on unstained samples. Dead cells were excluded by staining with DAPI (1 µg/mL). Double positive cells were collected directly into lysis buffer (Buffer RLT Plus, Qiagen) + β-mercaptoethanol (1:100) and stored at −80 °C until RNA extraction.

### RNA extraction, library preparation, and sequencing

NSCs of Control and Sox5^null^ samples (*n* = 3 and *n* = 4 samples, respectively, 2 pooled animals for each sample) were subjected to total RNA extraction using RNeasy Plus Micro Kit (Qiagen) following instructions of the manufacturer.

Samples were sent to Novogene UK and quality was measured using the Qubit 3.0 fluorimeter for sample concentration and Agilent 5400 for fragment analysis. SMART-Seq V4 Ultra Low Input RNA kit for Sequencing 480 Rxns (Cat No. 634893) was used for efficient cDNA synthesis and library preparation (especially from low RNA inputs). The first step is First-Strand cDNA Synthesis, the RNA is reverse transcribed into cDNA using SMART technology, which ensures full-length cDNA synthesis. The second step is cDNA Amplification, synthesized cDNA is then amplified using Long-Distance PCR (LD PCR) to generate sufficient material for sequencing. After this the amplified cDNA is purified using AMPure XP beads to remove any contaminants and ensure high-quality cDNA, which was quantified with Qubit.

The cDNA samples were then fragmented, end-repaired, A-tailed, and ligated with adaptors. After size selection and PCR enrichment, the RNA library was checked with Qubit and real-time PCR for quantification and bioanalyzer for size distribution detection. Quantified libraries were pooled and sequenced on Illumina platforms, according to effective library concentration and data amount. Samples were sequenced on the Illumina Novaseq X plus and mRNA-seq was sequenced using a PE150 (pair-end 150 base pair) strategy to produce 6G of data.

### RNA-Seq and differential expression analysis

Next-generation sequencing (NGS) data bioinformatic analysis has been performed by the Laboratory of Omics and Bioinformatics at Instituto Cajal (CSIC) Madrid, Spain. Quality analyses were performed over reads using FastQC1 (v0.11.8) software. The quality by position (Phred+33 quality score) maintained in general good standard across all cycles with median and mean base quality over 28. Reference genome and the annotation file of *Mus musculus* (GRCm39, mm39) have been downloaded from UCSC ftp site and GENECODE respectively. The reads were aligned against *M. musculus* genome using Hisat24 (v2.1.0) aligner. Before the alignment, the FASTQ files from the same sample, but generated each from different sequencing runs, were merged. Results showed a good behavior of reads in the alignment process, on average more than 96% of reads mapped against the reference genome.

Integrative Genomics Viewer [49] was used to visualize aligned reads and normalized coverage tracks **[**RPM (reads per million)]. We used htseq-count7 (v0.11.2) to count the reads mapping each feature. We have used the “intersection-strict” resolution mode, where reads are counted only if they are inside a gene or inside the exons of a gene. The differential expression analysis was performed using Deseq2, an R software package, and differentially expressed genes (DEG, with adjusted *p*-value < 0.05) were represented in heat map graph and volcano plot. Specific heat maps of signaling pathways of interest were also generated using the same Bioconductor package. RNA-Seq data reported in this study are accessible through the ENA accession number PRJEB85952.

### Gene set enrichment and pathway analyses

Identification of enriched biological functions and processes in DEGs was performed using gene ontology (GO), KEGG and PANTHER pathways databases using the R package clusterProfiler and the freely accessible Panther software. We analyzed GO terms for cellular processes, cellular components, and molecular functions, as well as overrepresented signaling pathways in our DEGs. We used the PANEV software (Pathway Network Visualizer) [35], which consists of a set of R packages for the visualization of gene set/pathway-based networks. This software is based on information available in the KEGG database and visualizes genes within a network of interconnected upstream and downstream pathways at multiple levels (from level 1 to n). Network visualization helps interpret the functional profiles of a group of genes. We used this software to construct networks of cell cycle, Wnt, and TGF-β/BMP signaling pathways.

### RNA extraction, cDNA synthesis, and real-time quantitative PCR

Total RNA of Control and Sox5^null^ neurospheres of P14 mice was extracted using the QuickGene RNA tissue kit S (Kurabo) and then treated with DNAse. cDNA was synthesized using First-Strand cDNA Synthesis kit (NZYtech). Gene expression levels were measured using TaqMan Gene expression assays (Applied Biosystems) and quantitative real-time PCR (RT-qPCR) was carried out in a QuantStudio3 System (Applied Biosystems) using NZYSupreme qPCR Probe Master Mix (NZYtech). The following Taqman assays probes were used: Anapc (Mm00614339_m1), Axin2 (Mm00443610_m1), Bmp2 (Mm01340178_m1), Bmp3 (Mm00557790_m1), Bmp7 (Mm00432102_m1), Bmpr1b (Mm03023971_m1), Ccna2 (Mm00438063_m1), Ccnb1 (Mm03053893_gH), Ctdsp1 (Mm00778482_s1), Ctdsp2 (Mm01254394_m1), Fstl5 (Mm00618418_m1), Gapdh (Mm99999915_g1), Huwe1 (Mm00615533_m1), Id4 (Mm00499701_m1), Mcm4 (Mm00725863_s1), Myc (Mm00487804_m1), Ppm1a (Mm00725963_s1), Ppm1h (Mm00620945_m1), Smurf2 (Mm03024086_m1), Sox5 (Mm01264584_m1) and Ythdf2 (Mm00661925_m1). Gene expression was measured relative to endogenous control Gapdh (Mm99999915_g1) and normalized to the expression of the Control sample in each group using the 2^−ΔΔCt^ method, as indicated in the corresponding figure. At least three independent neurosphere cultures of Control and Sox5^null^ mice were performed for each gene and samples were run in triplicates.

### NSCs culture and in vitro treatments

Postnatal P14 hippocampal NSCs were cultured as previously described [26]. Briefly, mice were euthanized with CO_2_, their brains were isolated, and the hippocampus were dissected, cut up into pieces and digested with Papain **[**0.66 mg/ml papain (Worthington) + 0.2 mg/ml cysteine (Sigma) + 0.2 mg/ml EDTA (Merck) + Hank’s buffer (Thermo Fisher)] for 15 min at 37 °C. After mechanical dissociation and washes with DMEM F12 (Thermo Fisher) to stop the reaction and washes with Hank’s, the disaggregated cell suspension was plated into MW12 plates with basal media **[**DMEM F12 + 1× N2 supplement (100×; Thermo Fisher) + 1X B27 supplement (50×; Thermo Fisher)], 20 ng/ml EGF (100 ng/ μl; PeproTech) and 20 ng/ml FGF2 (100 ng/ μl; PeproTech). Cells were incubated at 37 °C and 5% CO_2_. Normally, one single brain was used to prepare the culture and 4 wells of MW12 per brain were used. For hippocampal floating neurospheres, 20 ng/ml EGF and 20ng/ml FGF2 were added daily and were passaged by mechanical procedures and used from passage 0 until passage 15 for different cell treatments. For immunostaining experiments in intact neurospheres, we used Matrigel-coated glasses to attached grown neurospheres during 10–15 min at 37 °C. To prepare matrigel-coated glass, clean glass coverslips, were coated with diluted Matrigel (Thermo Fisher) in DMEM F12 (1:100) in MW24 and incubated 4–12 h at 37 °C.

To induce quiescence, cells were plated in basal media for 24 h and treated with 20 ng/ml FGF2 alone or in combination with 30 ng/ml recombinant mouse BMP4 (PeproTech). For BMP signaling inhibition experiments, NSCs were treated with 250 ng/ml Noggin (PeproTech) or 0.5 µM LDN193189 (MedChemExpress) for 72 h and then fixed with 4% PFA (Merck) on ice for 25 min. For the analysis of fluorophores retention, neurospheres were dissociated into a single cell suspension and incubated in 0.5 mL of PBS with 2 mg/ml Cell Trace Oregon Green 488 Carboxy- DFFDA SE (Thermo Fisher) for 7 min at 37 °C in the dark as previously described [12]. After that, cells were washed with BSA 0.5%, centrifuged at 200*g* for 10 min, resuspended and seeded in NSC complete medium. After 6 days, neurospheres were dissociated and the fluorescence intensity of cell tracer was measured in a flow cytometer (BD).

### Microscopic analysis and cell counting

All images were taken with a direct SP5 confocal microscope (Leica). Images of both left and right dorsal DG sections (−0.82 mm to −4.16 mm from bregma) were captured with a z-step of 2 μm through at least 20 μm of each 50 μm sections. Labeled cells were counted in the SGZ of every ninth of 50 µm DG sections. In Control and Sox5^null^ mice, the analysis was done counting the number of cells (at least 300 cells/marker) that expressed a cell-type-specific maker in the population of cells among SGZ cells expressing a certain cell-type marker. In Control and Sox5^icKO^ mutant mice, we counted recombined TdTom^+^ cells that were positive for the indicated marker. In those cases, 3–6 sections from at least 3–7 mice and a minimum of 100 cells for each animal were analyzed. Counting was performed manually and blind using LAS X (Leica) software. In all the cultures, three independent experiments were performed for each condition.

For quantification of fluorescence intensity, mean intensity was calculated using Fiji Image J Software.

### Statistical analysis

The appropriate sample size (N) was determined based on similar published data from other groups [10], using a minimum of 3 mice per condition for in vivo experiments, and a minimum of biological triplicate for in vitro experiments. Statistical analysis and graphs were conducted with GraphPad Prism version 8 software using different tests with a significance level of at least *p* < 0.05. Thus, two-tailed unpaired Student *t* test were used for most of statistical comparisons of two conditions for in vivo experiments; paired *t* test for in vitro experiments where control and treatment conditions for each biological replicate were performed in parallel. Statistical details were included in each figure and figure legend **[**number of experiments (*N*), number of cells (*n*), and statistical test]. Data are presented as mean ± SEM. Significance is stated as follows: *p* < 0.05 (*), *p* < 0.01 (**), *p* < 0.001 (***), confidence intervals of 95%.

### Drawings

All the drawings in the majority of figures have been done using SciDraw, except for the drawings in Fig 6I and S6B Fig that have been done using Inkscape 1.3.2.

### Ethics statement

Animal procedures were carried out in accordance with the guidelines of European Union (2010/63/UE), Spanish legislation (53/2013, BOE no. 1337) and under the approved projects (PROEX 078/17 and PROEX 146.0/22) reviewed by “Area de Protección Animal” from Dirección General de Agricultura, Ganadería y Alimentación, Comunidad de Madrid (Spain).

## Supporting information

S1 Fig Sox5 is expressed in Sox2^+^ progenitors and NSCs during DG development. **(A, B)** Confocal images showing Sox5 and Sox2 immunostaining in E16.5 (A) and P0 (B) mouse hippocampal sections. CA, Cornus Ammonis; CH, cortical hem; DG, dentate gyrus. **(C)** Confocal images showing Sox5^+^ cells in dorsal DG in P5 and P14 mice and in combination with the indicated markers in P5 mice. **(D, E)** Quantitation of positive cells for the indicated marker amongst Sox5^+^ (D) or Sox2^+^ population (E) in P5 and P14 mice. At least three animals and three sections/animal were analyzed for each immunostaining. In all graphs, data are mean value ± SEM. ***p < 0.001 by unpaired Student *t* test. Scale bar represents 100 µm (A left, B right), 150 µm (B left, C left), and 25 µm (A right, F right). Data are available in S1 Data as a part of Supporting information.
(TIF)

S2 Fig Loss of Sox5 expression causes an early reduction in neurogenesis by P14. **(A)** Confocal images showing DCX and BrdU immunostaining in the SGZ of DG at the indicated stages in Control and Sox5^null^ mice. **(B)** Scheme of BrdU labeling of newborn neurons from P12 to P14. **(C)** Quantitation of DCX^+^ BrdU^+^ cells/mm^2^ in P14 Control and Sox5^null^ mice. In the graph, data are mean value ± SEM. *p < 0.05 by unpaired Student *t* test. Scale bar represents 25 µm in A. Data are available in S1 Data as a part of Supporting information. Drawing was created through SciDraw.
(TIF)

S3 Fig Fluorescent Activated Cell Sorting (FACS)-based strategy to enrich for NSCs from P14 Control and Sox5^null^ mice DGs. **(A)** Scheme of the NSCs enrichment procedure using anti-GLAST-PE and anti-Prom1-APC antibodies and FACS-sorting. **(B, D)** FACS histograms for dissociated P14 Control (B) and Sox5^null^ (D) DG indicating the sorted double positive GLAST^+^Prom^+^, 12.36% and 13.63%, respectively, of live (DAPI^−^), single cells. **(C)** FACS histograms for dissociated P14 Control mice DG after the incubation with anti-GLAST or anti-Prom1 antibodies. **(E)** Quantitation of the relative levels of Sox2 mRNA expression by quantitative PCR in Control GLAST^+^Prom^+^ and GLAST^−^Prom^−^ FACS-sorted cells indicated in (B). Results are shown as 2^−ΔΔCT^ normalized with respect to GAPDH mRNA and relative to GLAST^+^Prom^+^ values. Data represent mean values ± SEM. **(F)** Heat map of relative gene expression associated to the indicated DG cell type (astrocytes, RGLs/NSCs, IPCs, NB1 and NB2; gene list taken from Hochgerner and colleagues, 2018) after RNA-Seq of Control GLAST^+^Prom^+^ cell populations. Four control samples (each containing cells from DG two P14 mice, S2C to S7C) were independently FACS-sorted; total RNA was purified, amplified and libraries were prepared for bulk RNA deep sequencing (see Materials and methods). Data are available in S1 Data and S2 Data as a part of Supporting information. Drawings were created through SciDraw, Bioicons, Bioart, and Inkscape.
(TIF)

S4 Fig Transcriptomic analysis of Sox5^null^ NSCs reveals important changes in developmental programs including and downregulation of Wnt signaling pathways. **(A)** Node graphs including DEGs ascribed to Wnt signaling pathways by KEGG software and generated by the PANEV software PAthway NEtwork Visualizer v 17 0 from the bulk RNA-Seq analysis performed in FACS-sorted NSCs from P14 Sox5^null^ and Control mice.
(TIF)

S5 Fig Analysis of neurosphere number and diameter in P14 Control and Sox5^null^ mice DG in acute NSC preparation. **(A)** Acute NSCs preparation from P14 DG, from passage 0 (primary, 1^ry^) to passage 5. Quantitation of neurosphere number per area for 1^ry^ and 2^ry^cultures, and in successive passages for Control and Sox5^null^ mice. (B) Quantitation of neurosphere diameter in 1^ry^, 2^ry^, and 3^ry^ cultures for Control and Sox5^null^ mice. Data are mean value ± SEM. ***p < 0.001 by unpaired Student *t* test. Data are available in S1 Data as a part of Supporting information.
(TIF)

S6 Fig Noggin or BMPR inhibitor LDN193189 reduce the number of pSmad1/5/9^+^ NSCs in vitro. **(A)** Confocal images showing pSmad1/5/9 immunostaining of P14 NSCs in the indicated culture conditions. **(B)** Scheme of the BMP canonical signaling pathway highlighting the endogenous inhibitor Noggin and a small molecule pharmacological inhibitor LDN193189, which inhibits exclusively the kinase activity of ACVR1, BMPR1A, and BMPR1B. **(C)** Quantitation of the percentage of pSmad1/5/9^+^ cells relative to total cell number in Control P14 NSCs grown in quiescence conditions (FGF2 + BMP4) and treated with DMSO as control or the BMP pathway inhibitor LDN193189. Data represent mean values ± SEM. *p < 0.05 according to unpaired Student *t* test. Scale bars represent 30 µm (A). Data are available in S1 Data as a part of Supporting information. Drawings were created through Inkscape.
(TIF)

S1 Data Source data for main figures and supporting figures. (XLSX)

S2 Data FACS source data for main figures and supporting figures. (ZIP)

## Abbreviations

aNSC: active neural stem cell
BrdU-L: BrdU-long retaining NSCs
DEG: differentially expressed genes
DG: dentate gyrus
GN: granule neurons
IPC: intermediate progenitor cell
LDN193189: LDN
Nog: Noggin
NSC: neural stem cells
qNSC: quiescent neural stem cell
RT-qPCR: quantitative reverse transcription polymerase chain reaction
SGZ: subgranular zone
TAM: tamoxifen
