# Supplementary figures and images for: Sox5 controls the establishment of quiescence in neural stem cells during postnatal development
Source: PLoS Biol. 2025 Jul 28;23(7):e3002654. doi: 10.1371/journal.pbio.3002654 (PMC12321142; doi:10.1371/journal.pbio.3002654)

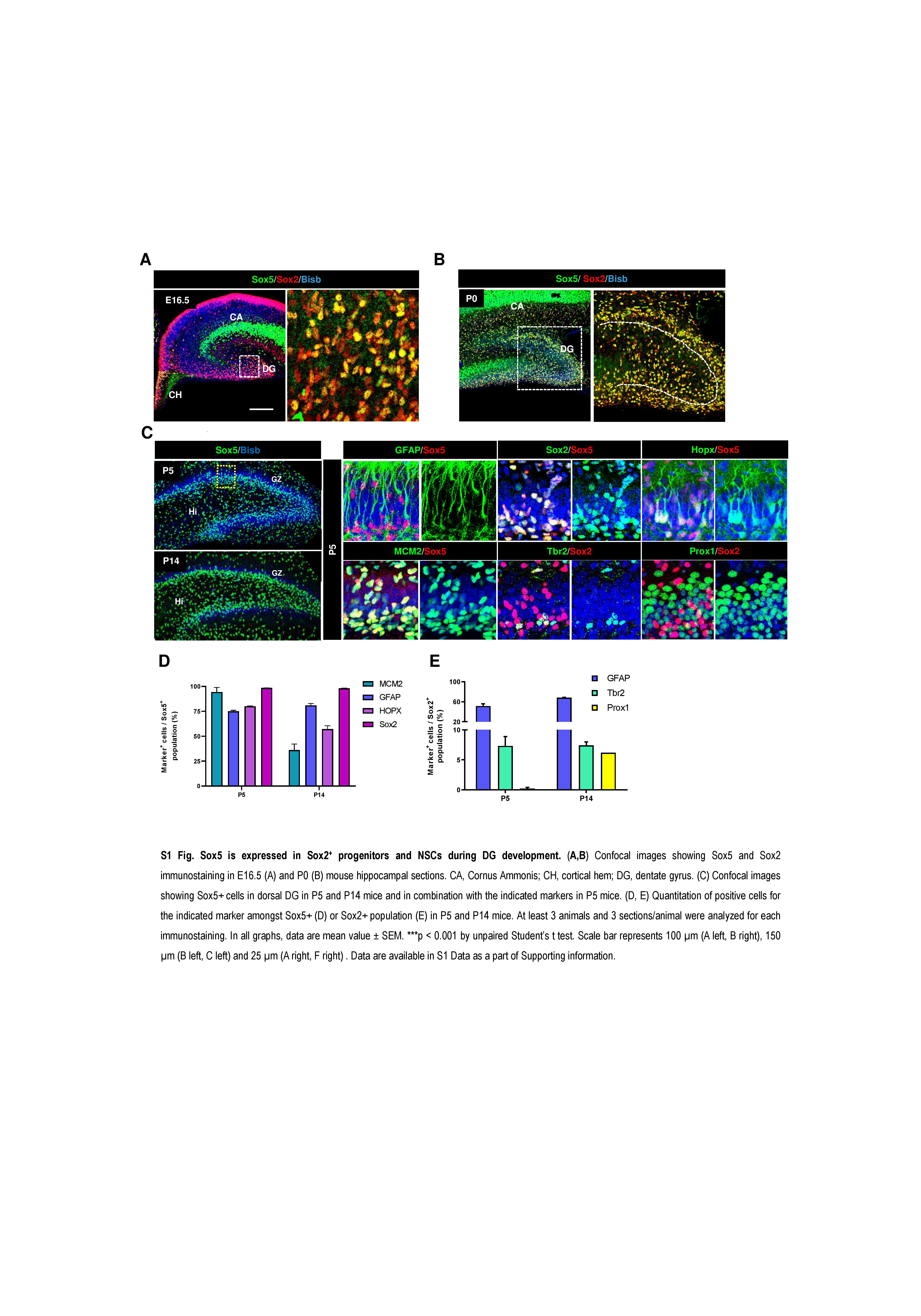

Supplement: S1 Fig — (A, B) Confocal images showing Sox5 and Sox2 immunostaining in E16.5 (A) and P0 (B) mouse hippocampal sections. CA, Cornus Ammonis; CH, cortical hem; DG, dentate gyrus. (C) Confocal images showing Sox5+ cells in dorsal DG in P5 and P14 mice and in combination with the indicated markers in P5 mice. (D, E) Quantitation of positive cells for the indicated marker amongst Sox5+ (D) or Sox2+ population (E) in P5 and P14 mice. At least three animals and three sections/animal were analyzed for each immunostaining. In all graphs, data are mean value ± SEM. ***p < 0.001 by unpaired Student t test. Scale bar represents 100 µm (A left, B right), 150 µm (B left, C left), and 25 µm (A right, F right). Data are available in S1 Data as a part of Supporting information. (TIF) [file pbio.3002654.s001.tif]

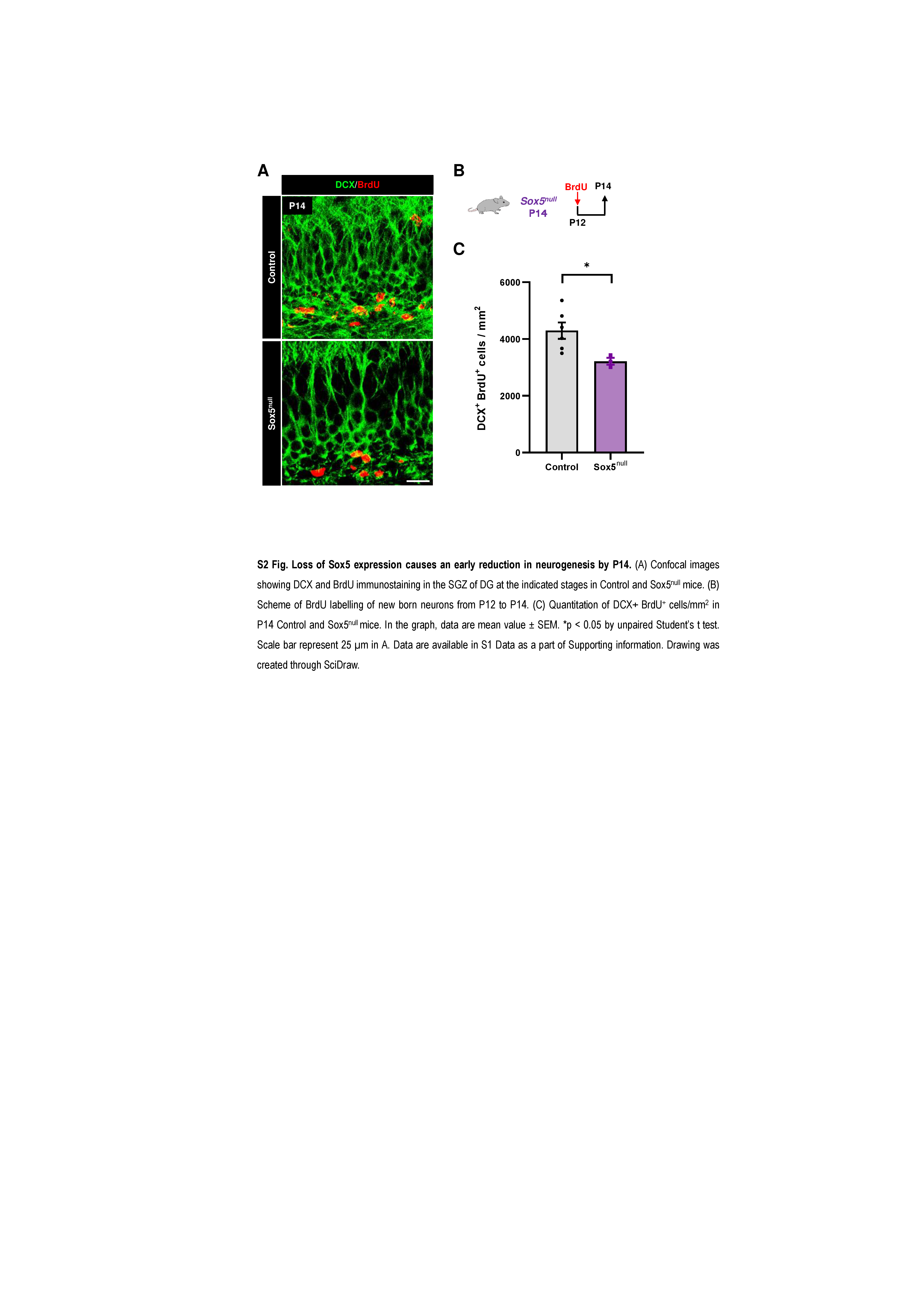

Supplement: S2 Fig — (A) Confocal images showing DCX and BrdU immunostaining in the SGZ of DG at the indicated stages in Control and Sox5null mice. (B) Scheme of BrdU labeling of newborn neurons from P12 to P14. (C) Quantitation of DCX+ BrdU+ cells/mm2 in P14 Control and Sox5null mice. In the graph, data are mean value ± SEM. *p < 0.05 by unpaired Student t test. Scale bar represents 25 µm in A. Data are available in S1 Data as a part of Supporting information. Drawing was created through SciDraw. (TIF) [file pbio.3002654.s002.tif]

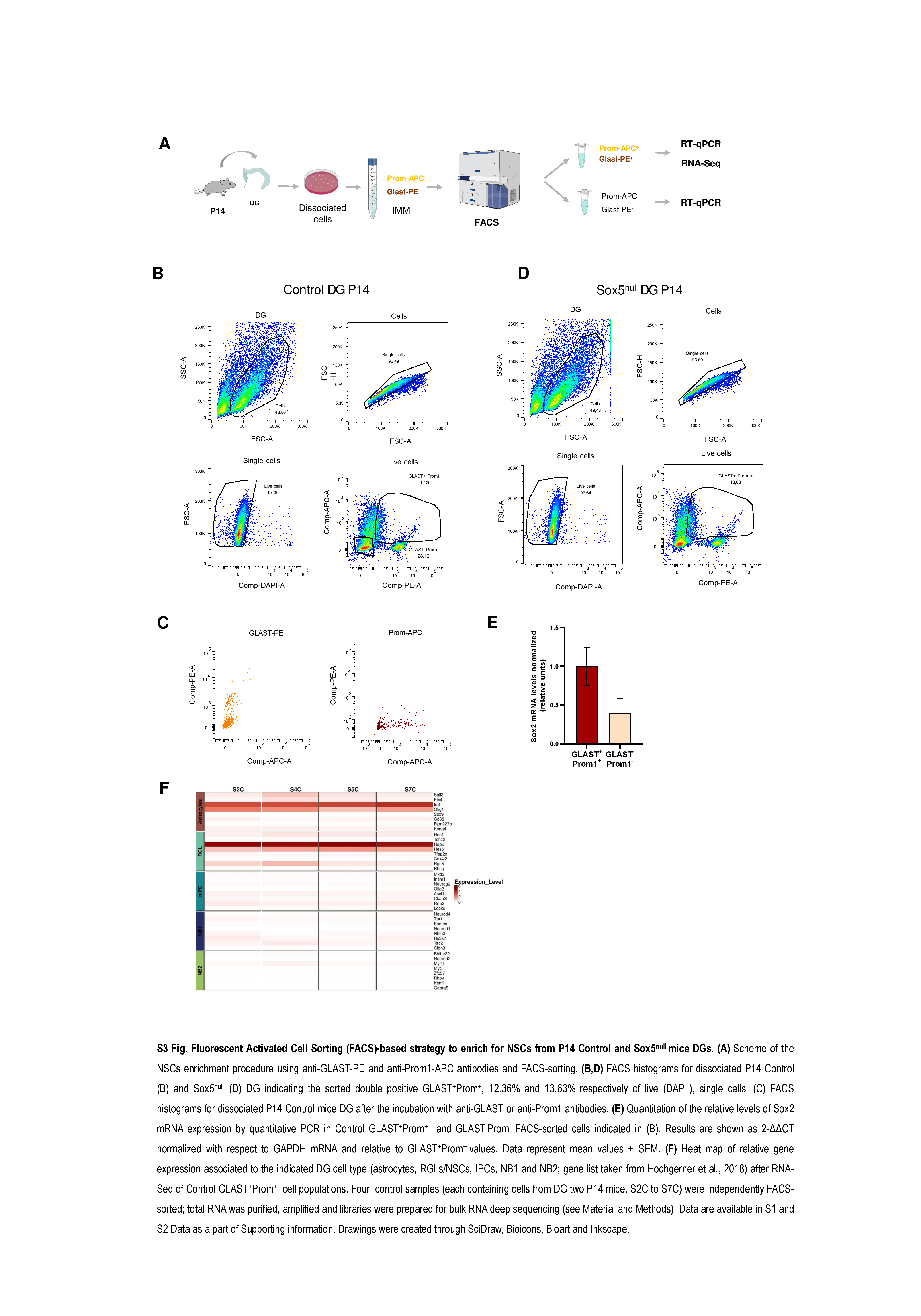

Supplement: S3 Fig — (A) Scheme of the NSCs enrichment procedure using anti-GLAST-PE and anti-Prom1-APC antibodies and FACS-sorting. (B, D) FACS histograms for dissociated P14 Control (B) and Sox5null (D) DG indicating the sorted double positive GLAST+Prom+, 12.36% and 13.63%, respectively, of live (DAPI−), single cells. (C) FACS histograms for dissociated P14 Control mice DG after the incubation with anti-GLAST or anti-Prom1 antibodies. (E) Quantitation of the relative levels of Sox2 mRNA expression by quantitative PCR in Control GLAST+Prom+ and GLAST−Prom− FACS-sorted cells indicated in (B). Results are shown as 2−ΔΔCT normalized with respect to GAPDH mRNA and relative to GLAST+Prom+ values. Data represent mean values ± SEM. (F) Heat map of relative gene expression associated to the indicated DG cell type (astrocytes, RGLs/NSCs, IPCs, NB1 and NB2; gene list taken from Hochgerner and colleagues, 2018) after RNA-Seq of Control GLAST+Prom+ cell populations. Four control samples (each containing cells from DG two P14 mice, S2C to S7C) were independently FACS-sorted; total RNA was purified, amplified and libraries were prepared for bulk RNA deep sequencing (see Materials and methods). Data are available in S1 Data and S2 Data as a part of Supporting information. Drawings were created through SciDraw, Bioicons, Bioart, and Inkscape. (TIF) [file pbio.3002654.s003.tif]

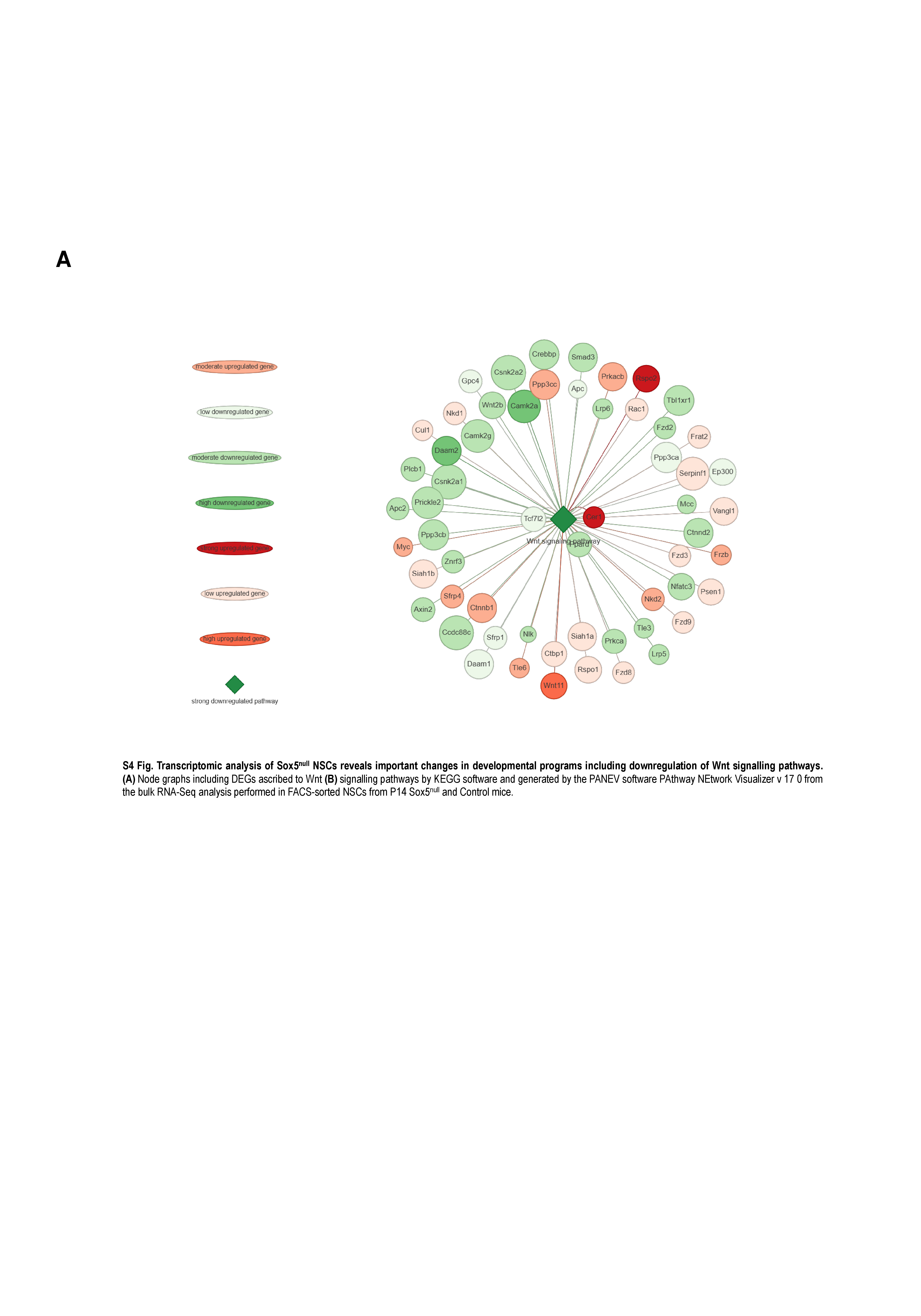

Supplement: S4 Fig — (A) Node graphs including DEGs ascribed to Wnt signaling pathways by KEGG software and generated by the PANEV software PAthway NEtwork Visualizer v 17 0 from the bulk RNA-Seq analysis performed in FACS-sorted NSCs from P14 Sox5null and Control mice. (TIF) [file pbio.3002654.s004.tif]

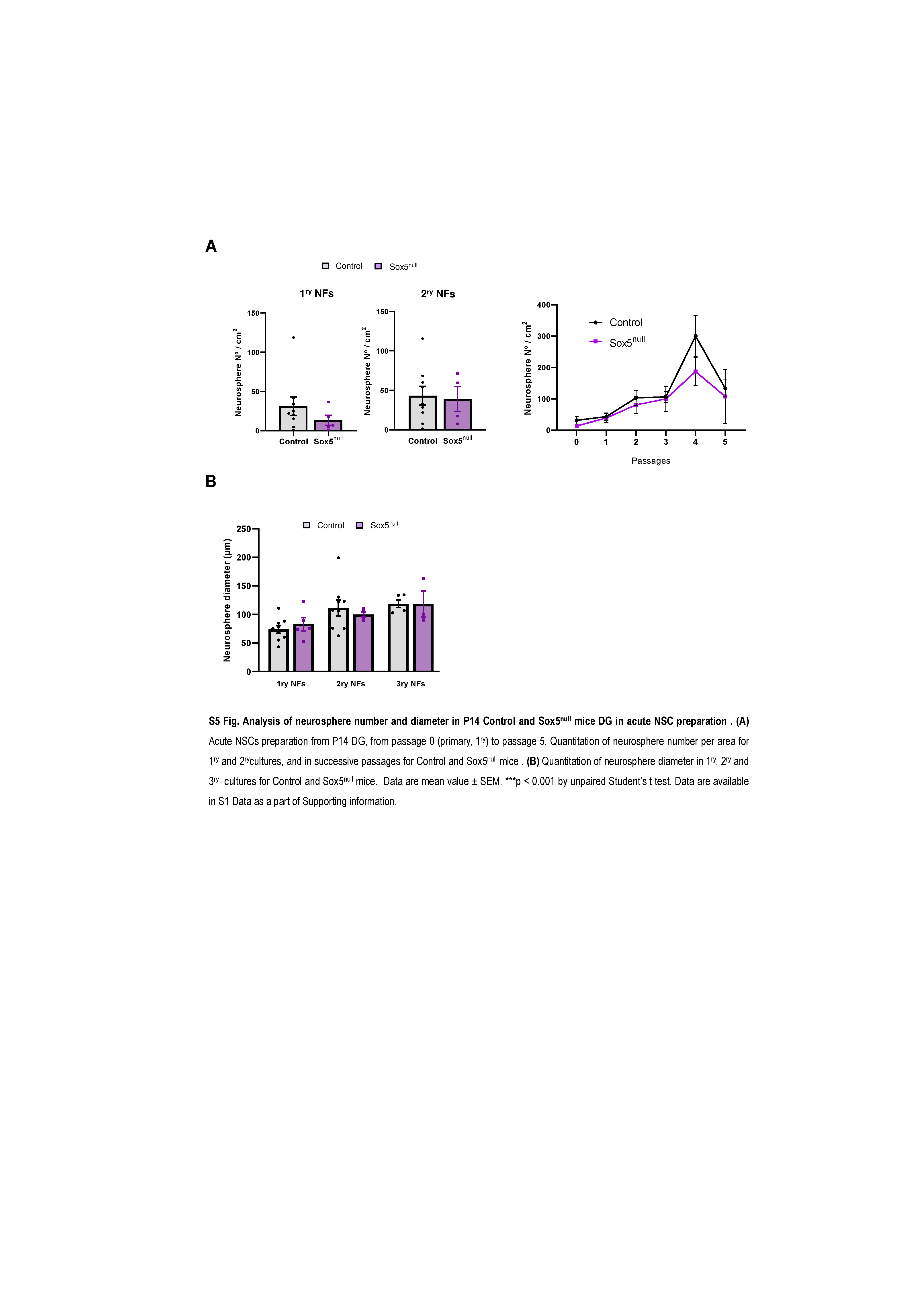

Supplement: S5 Fig — (A) Acute NSCs preparation from P14 DG, from passage 0 (primary, 1ry) to passage 5. Quantitation of neurosphere number per area for 1ry and 2rycultures, and in successive passages for Control and Sox5null mice. (B) Quantitation of neurosphere diameter in 1ry, 2ry, and 3ry cultures for Control and Sox5null mice. Data are mean value ± SEM. ***p < 0.001 by unpaired Student t test. Data are available in S1 Data as a part of Supporting information. (TIF) [file pbio.3002654.s005.tif]

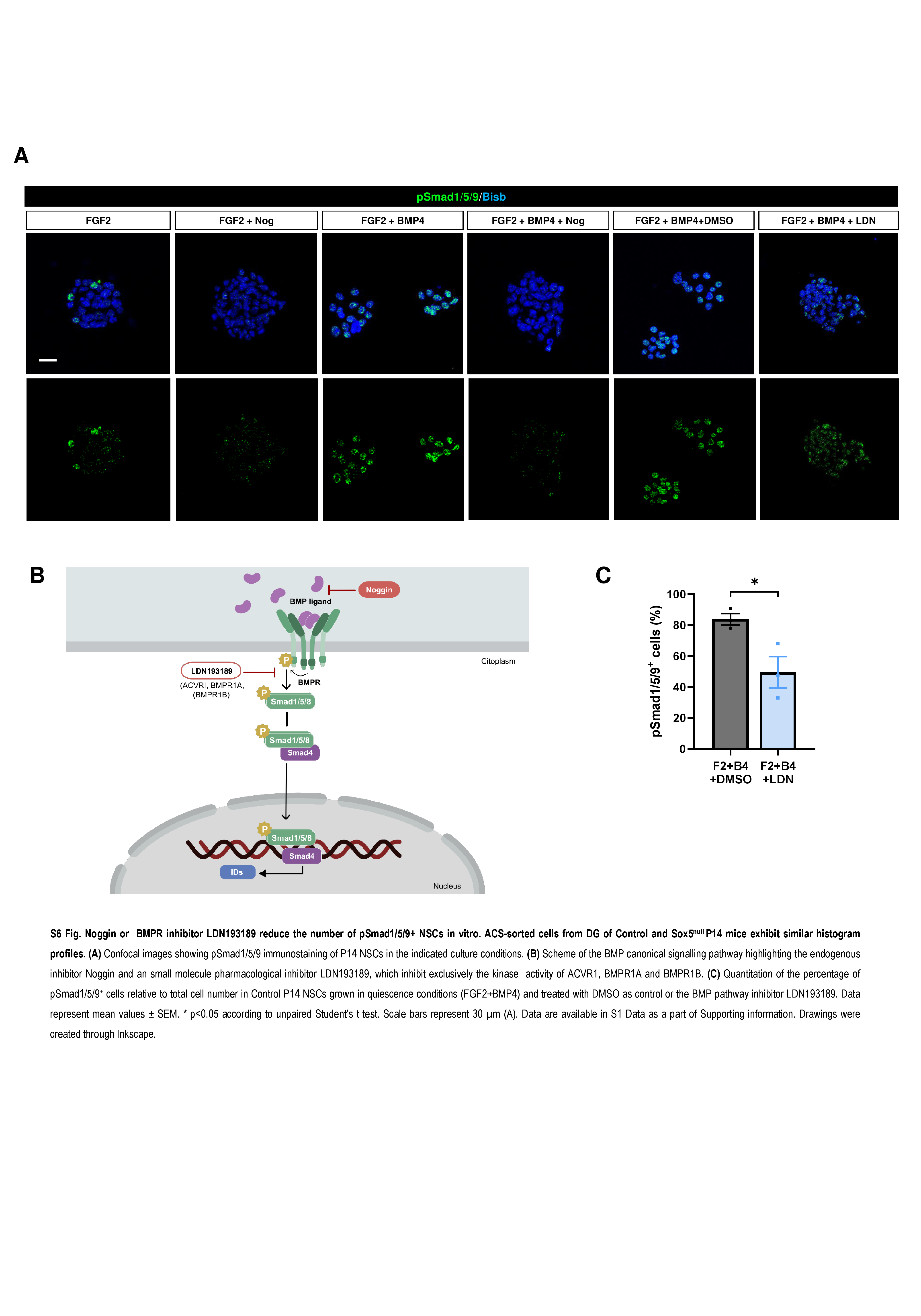

Supplement: S6 Fig — (A) Confocal images showing pSmad1/5/9 immunostaining of P14 NSCs in the indicated culture conditions. (B) Scheme of the BMP canonical signaling pathway highlighting the endogenous inhibitor Noggin and a small molecule pharmacological inhibitor LDN193189, which inhibits exclusively the kinase activity of ACVR1, BMPR1A, and BMPR1B. (C) Quantitation of the percentage of pSmad1/5/9+ cells relative to total cell number in Control P14 NSCs grown in quiescence conditions (FGF2 + BMP4) and treated with DMSO as control or the BMP pathway inhibitor LDN193189. Data represent mean values ± SEM. *p < 0.05 according to unpaired Student t test. Scale bars represent 30 µm (A). Data are available in S1 Data as a part of Supporting information. Drawings were created through Inkscape. (TIF) [file pbio.3002654.s006.tif]
